# Supplementary figures and images for: DYRK1A interacts with the tuberous sclerosis complex and promotes mTORC1 activity
Source: eLife. 2024 Oct 22;12:RP88318. doi: 10.7554/eLife.88318 (PMC11495841; doi:10.7554/eLife.88318)

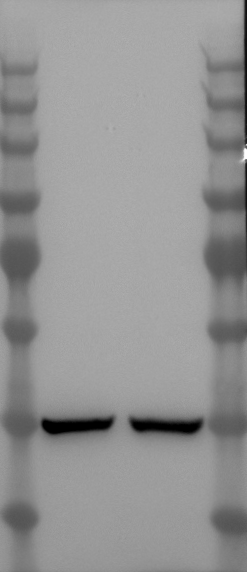

Supplement: Figure 1—source data 2. [file elife-88318-fig1-data2.zip › Figure1A-Actin.tif]

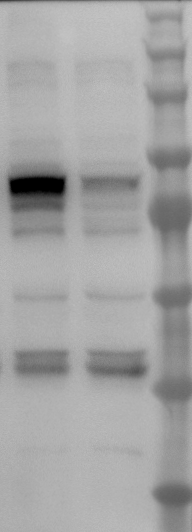

Supplement: Figure 1—source data 2. [file elife-88318-fig1-data2.zip › Figure1A-DYRK1A.tif]

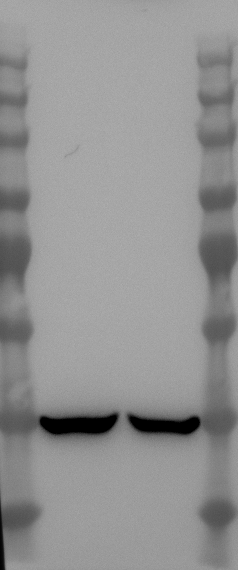

Supplement: Figure 1—source data 4. [file elife-88318-fig1-data4.zip › Figure1C-Act.tif]

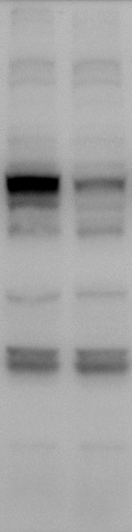

Supplement: Figure 1—source data 4. [file elife-88318-fig1-data4.zip › Figure1C-DYRK1A.tif]

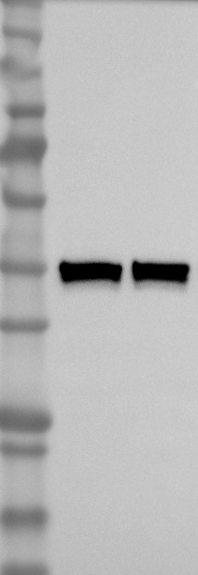

Supplement: Figure 1—source data 6. [file elife-88318-fig1-data6.zip › Figure1E-Act.tif]

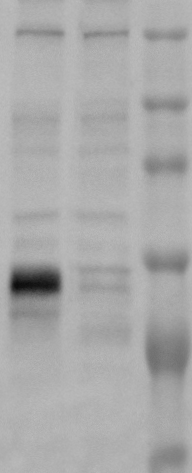

Supplement: Figure 1—source data 6. [file elife-88318-fig1-data6.zip › Figure1E-DYRK1A.tif]

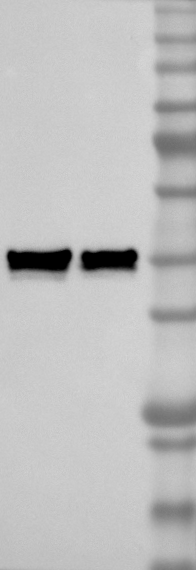

Supplement: Figure 2—source data 2. [file elife-88318-fig2-data2.zip › Figure2B-Actin.tif]

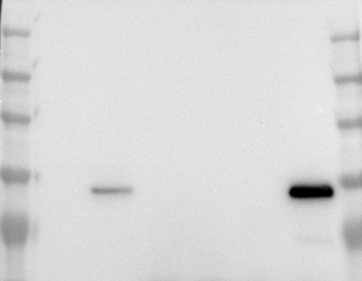

Supplement: Figure 2—source data 2. [file elife-88318-fig2-data2.zip › Figure2B-Flag(DYRK1A).tif]

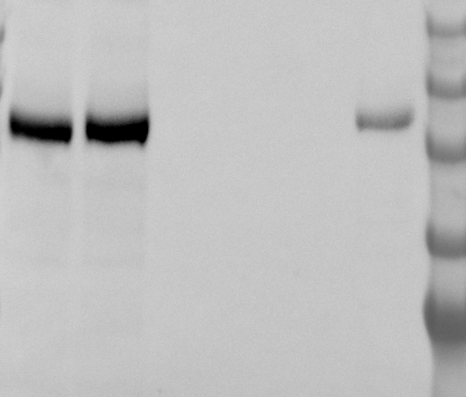

Supplement: Figure 2—source data 2. [file elife-88318-fig2-data2.zip › Figure2B-TSC1.tif]

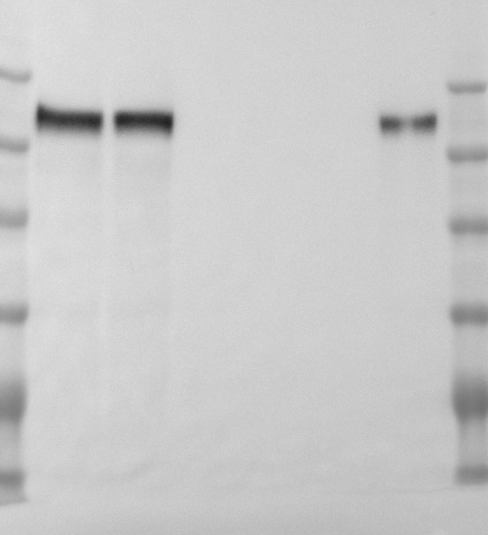

Supplement: Figure 2—source data 2. [file elife-88318-fig2-data2.zip › Figure2B-TSC2-full.tif]

## Slide 1
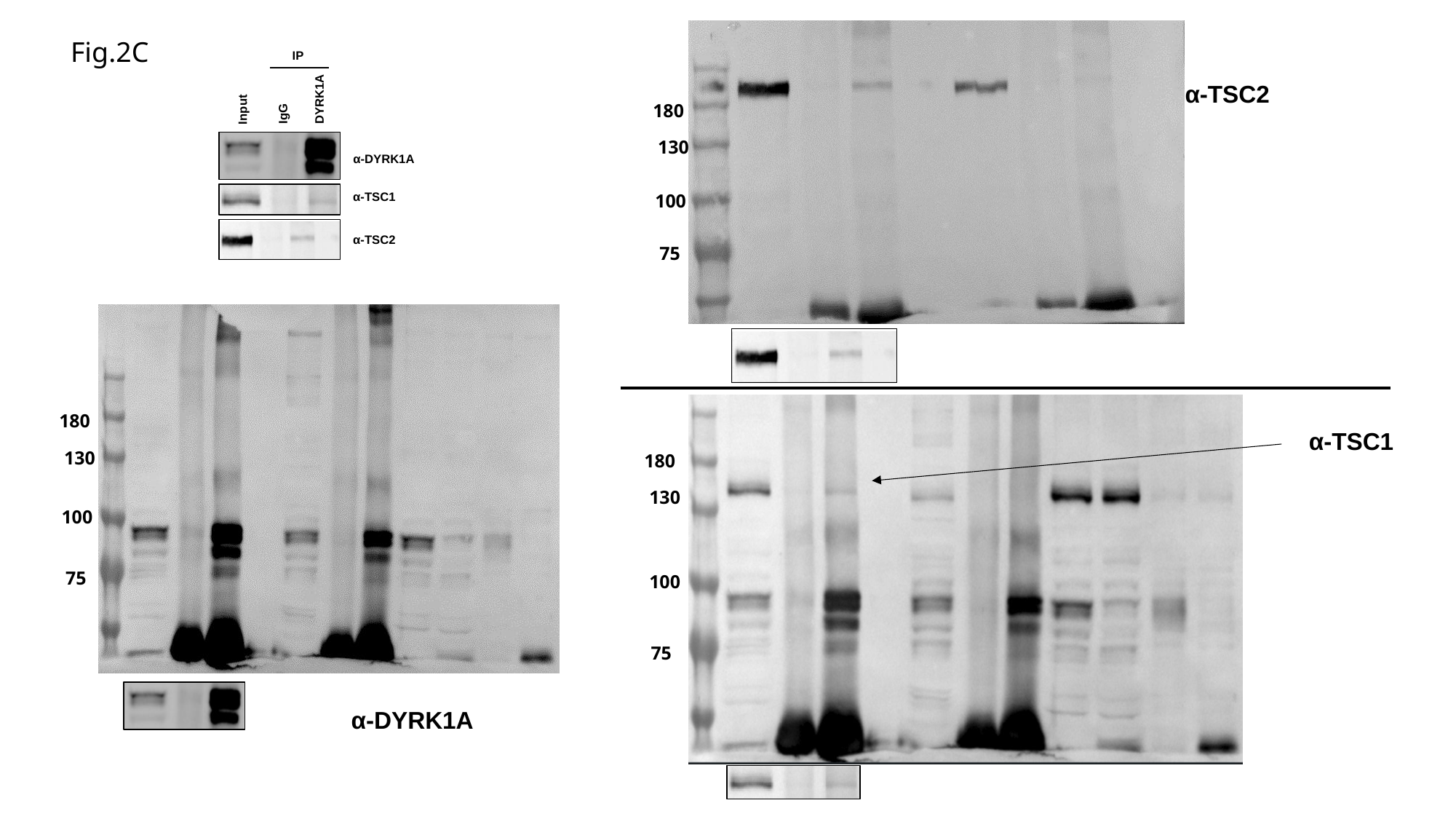

Fig.2C
IP
DYRK1A
IgG
Input
α-DYRK1A
α-TSC1
α-TSC2
α-TSC2
180
130
100
75
180
α-TSC1
130
180
130
100
75
100
75
α-DYRK1A

Supplement: Figure 2—source data 3. [file elife-88318-fig2-data3.zip › Figure 2C-source data.pptx]

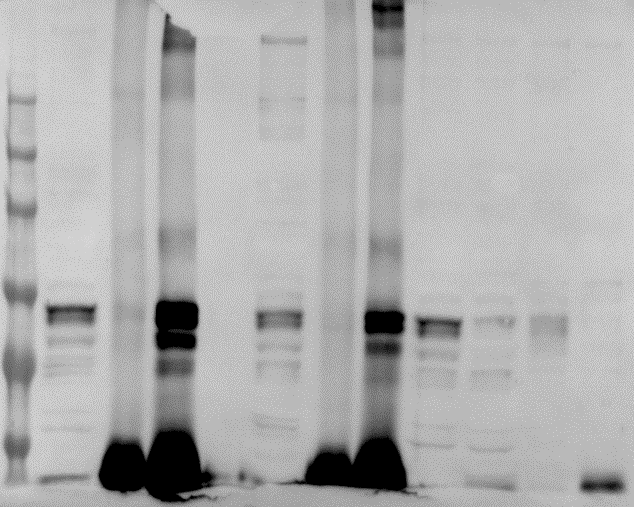

Supplement: Figure 2—source data 4. [file elife-88318-fig2-data4.zip › Figure2C-DYRK1A.tif]

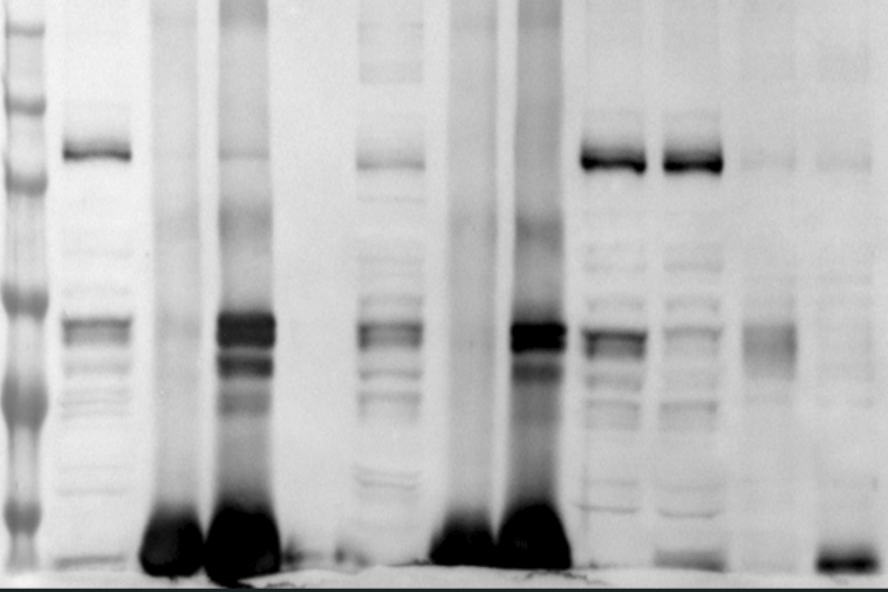

Supplement: Figure 2—source data 4. [file elife-88318-fig2-data4.zip › Figure2C-TSC1.tif]

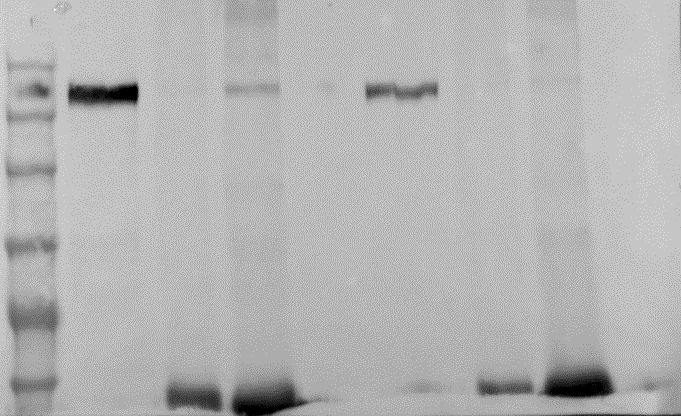

Supplement: Figure 2—source data 4. [file elife-88318-fig2-data4.zip › Figure2C-TSC2.tif]

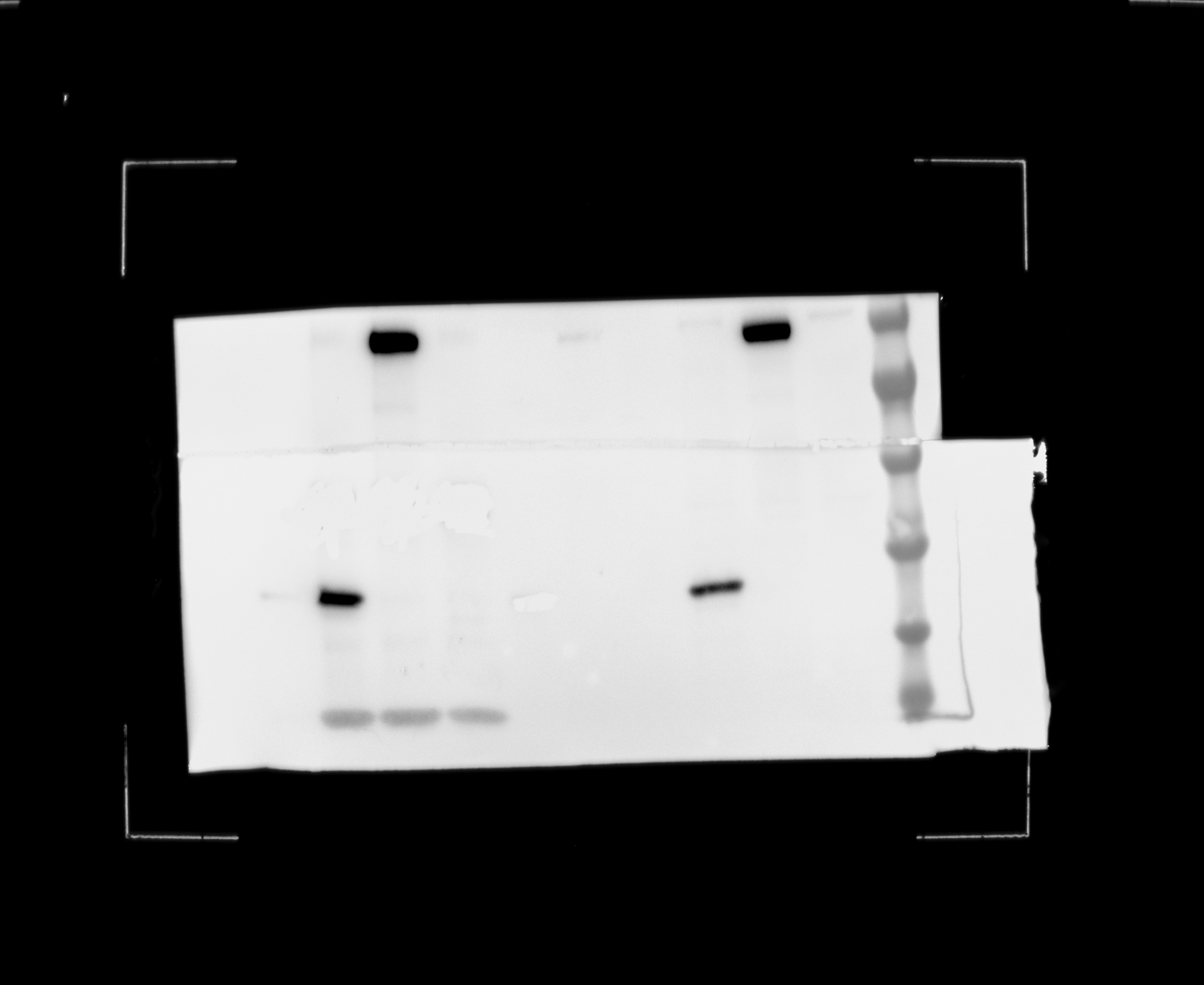

Supplement: Figure 2—source data 6. [file elife-88318-fig2-data6.zip › Figure2D-1-Flag.tif]

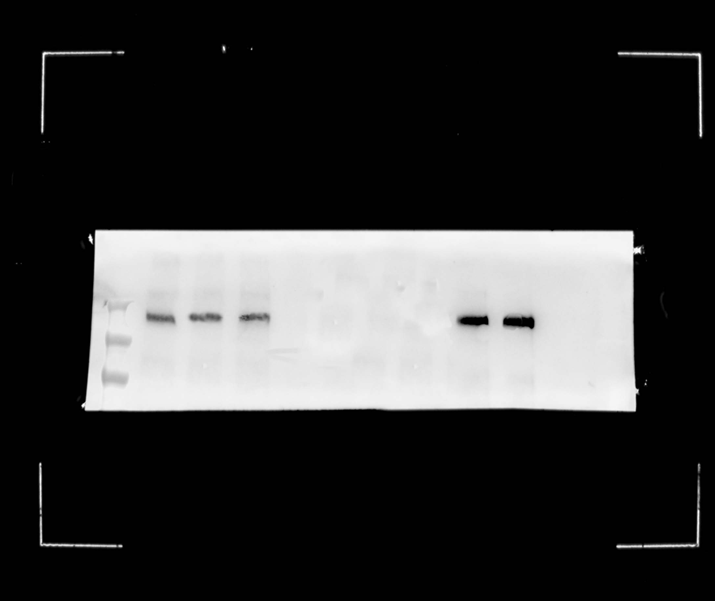

Supplement: Figure 2—source data 6. [file elife-88318-fig2-data6.zip › Figure2D-1-HA.png]

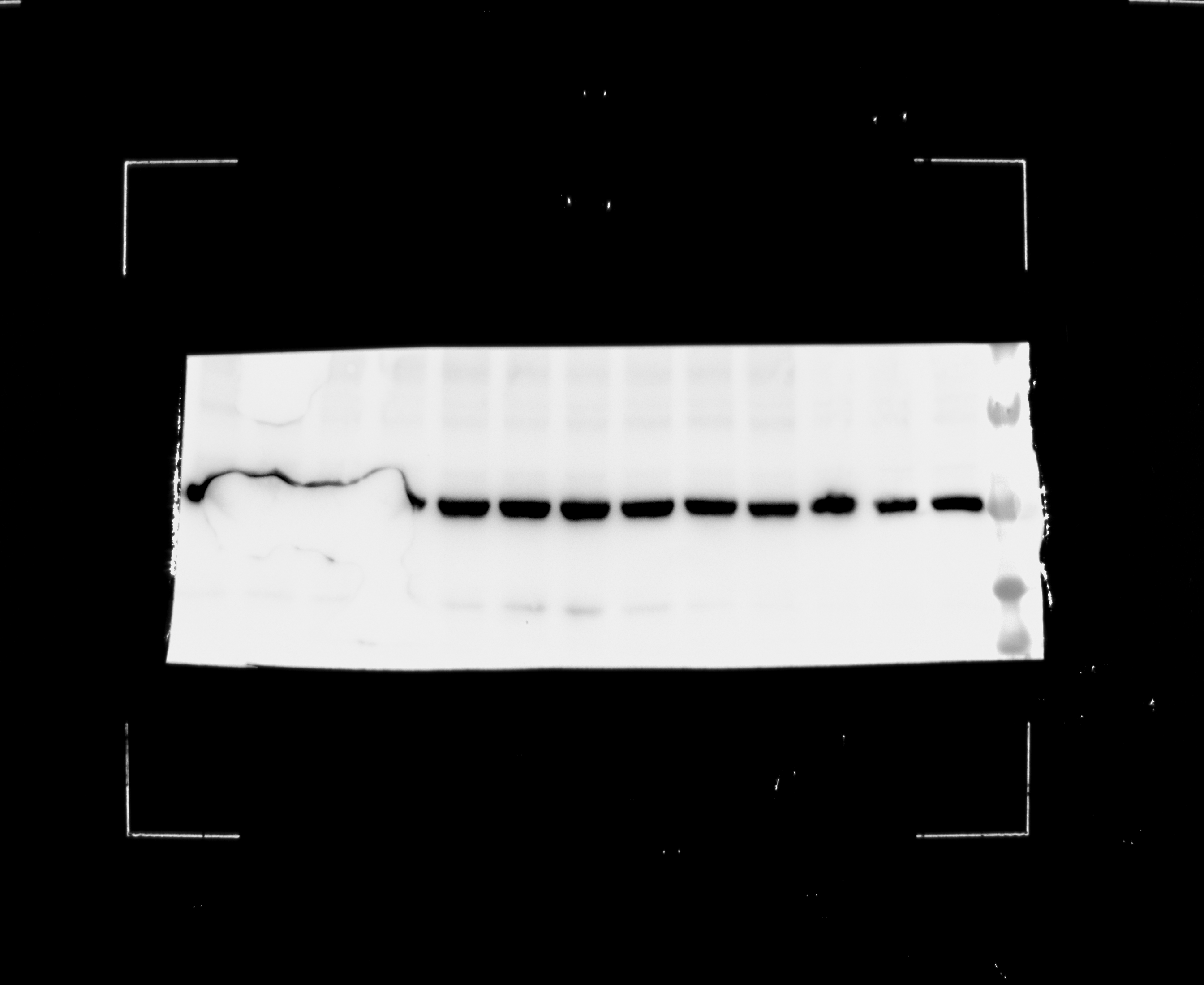

Supplement: Figure 2—source data 6. [file elife-88318-fig2-data6.zip › Figure2D-3-Actin.tif]

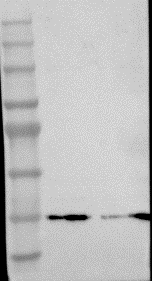

Supplement: Figure 2—figure supplement 1—source data 2. [file elife-88318-fig2-figsupp1-data2.zip › Fig2-Sup1-Actin.png]

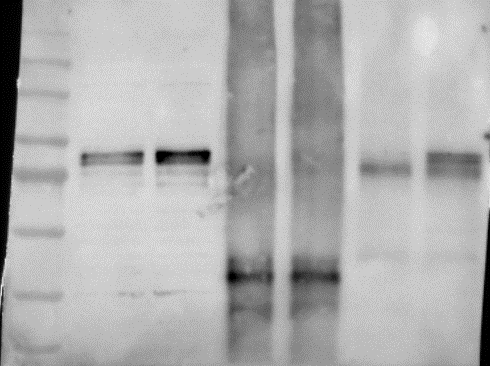

Supplement: Figure 2—figure supplement 1—source data 2. [file elife-88318-fig2-figsupp1-data2.zip › Fig2-Sup1-DYRK1A.png]

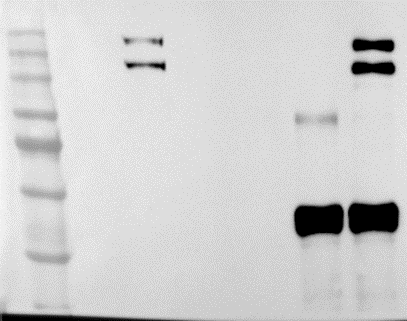

Supplement: Figure 2—figure supplement 1—source data 2. [file elife-88318-fig2-figsupp1-data2.zip › Fig2-Sup1-TSC1.png]

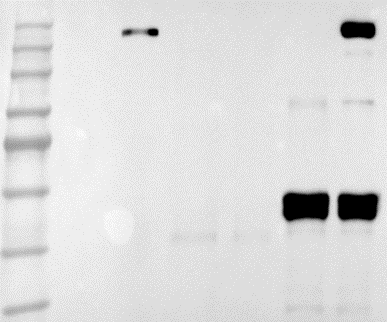

Supplement: Figure 2—figure supplement 1—source data 2. [file elife-88318-fig2-figsupp1-data2.zip › Fig2-Sup1-TSC2.png]

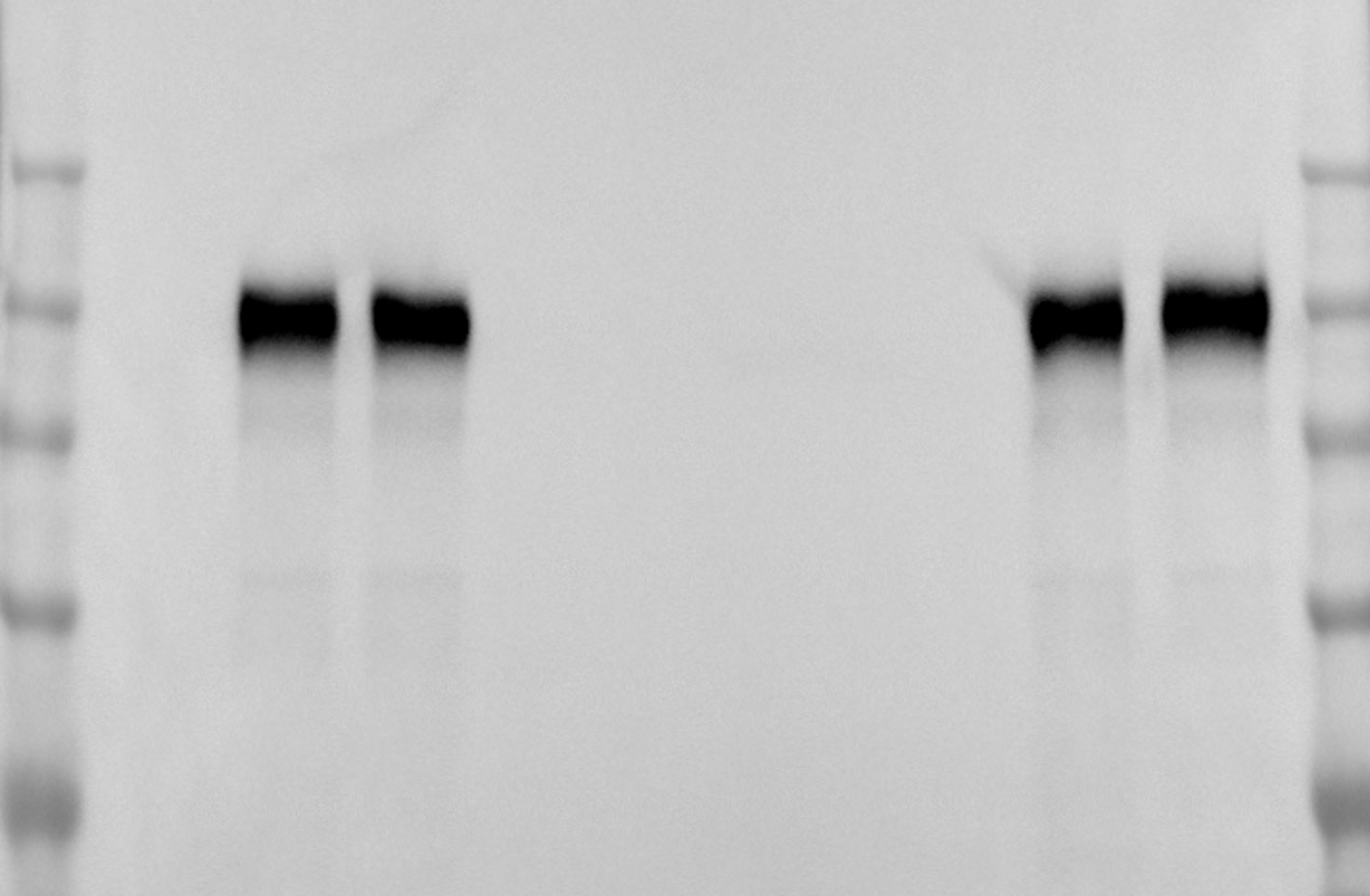

Supplement: Figure 2—figure supplement 2—source data 2. [file elife-88318-fig2-figsupp2-data2.zip › Fig2-Sup-HA-IP.tif]

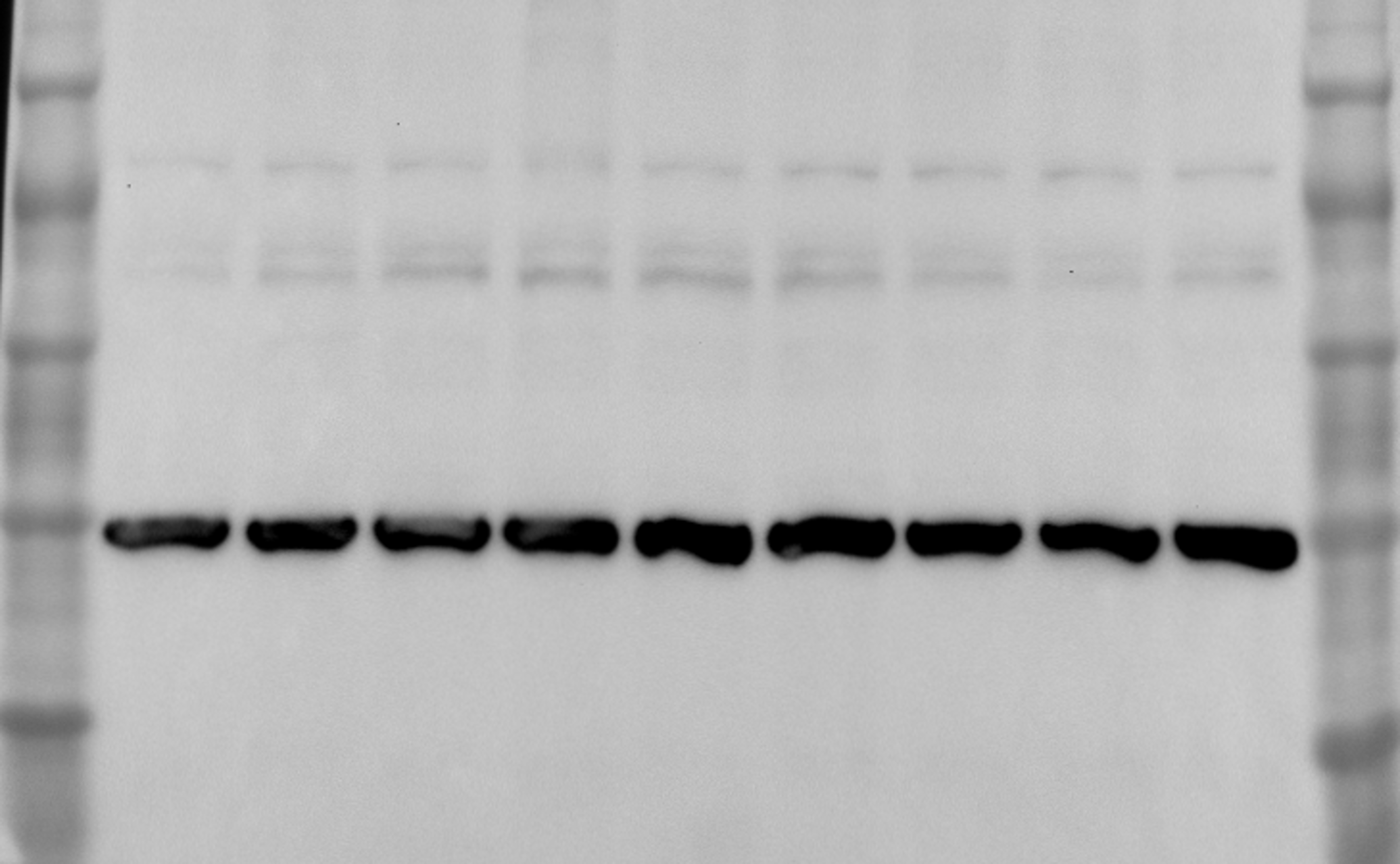

Supplement: Figure 2—figure supplement 2—source data 2. [file elife-88318-fig2-figsupp2-data2.zip › Fig2-Sup-Actin.tif]

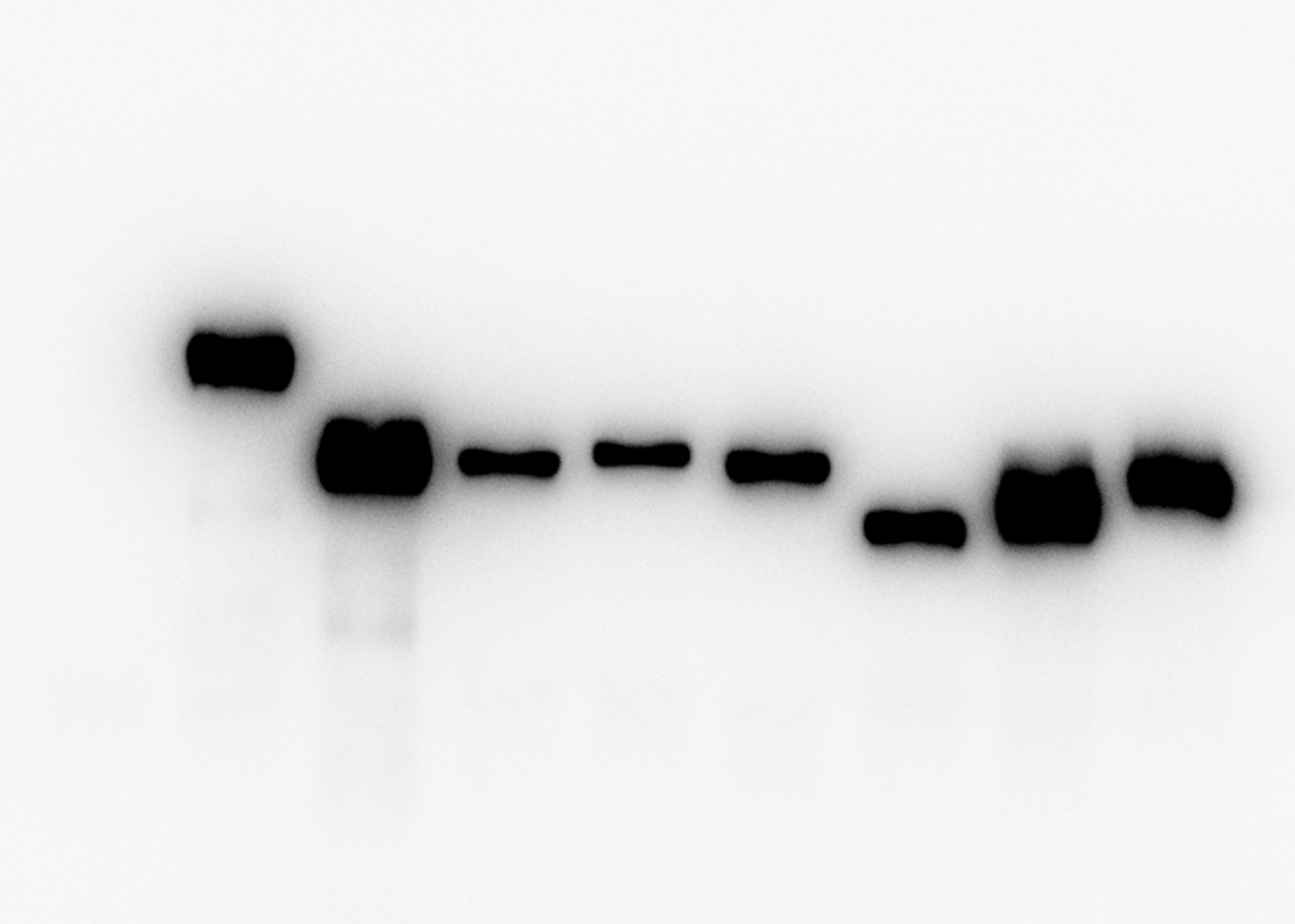

Supplement: Figure 2—figure supplement 2—source data 2. [file elife-88318-fig2-figsupp2-data2.zip › Fig2-Sup-Flag-IP.tif]

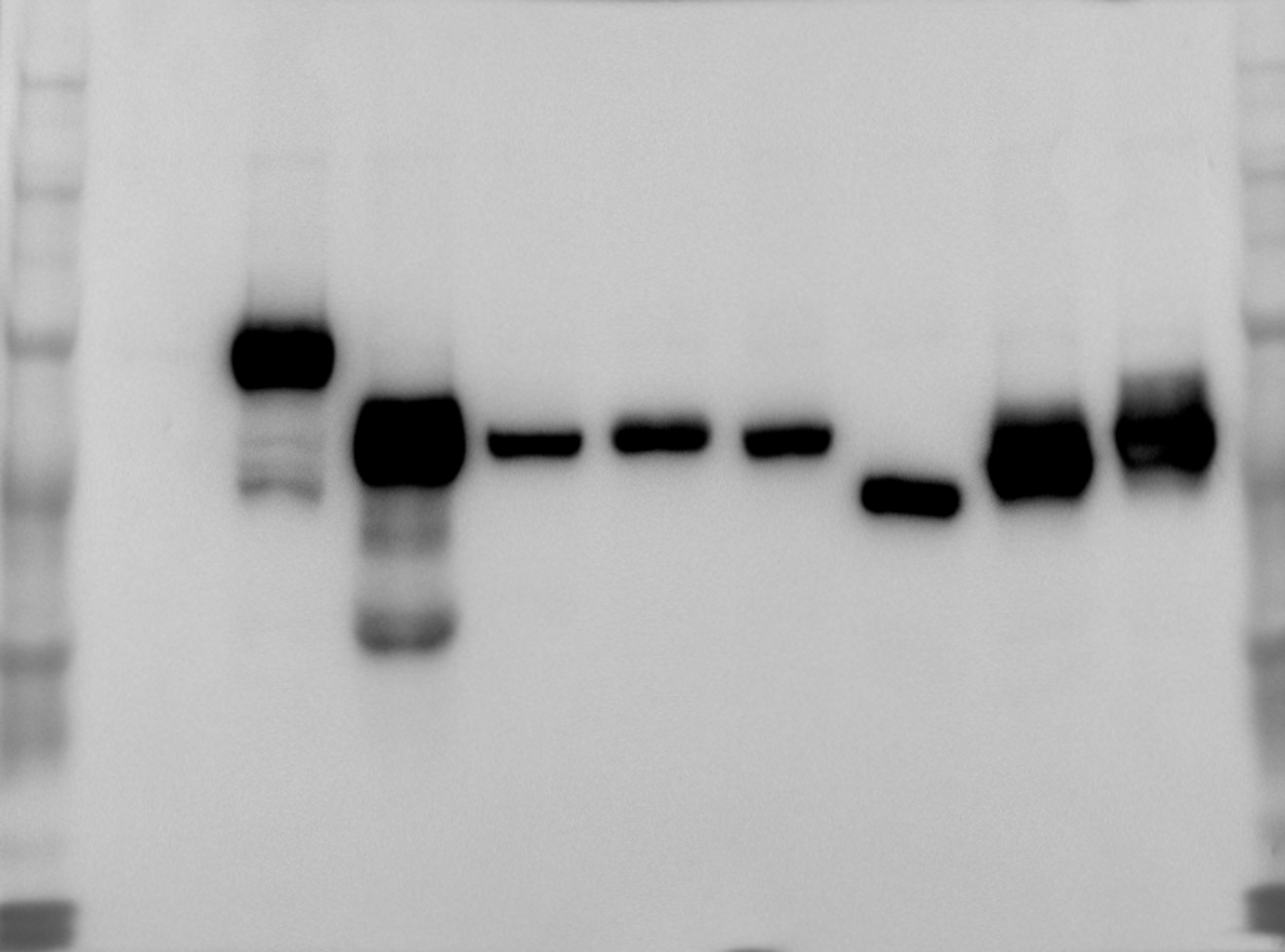

Supplement: Figure 2—figure supplement 2—source data 2. [file elife-88318-fig2-figsupp2-data2.zip › Fig2-Sup-Flaginput.tif]

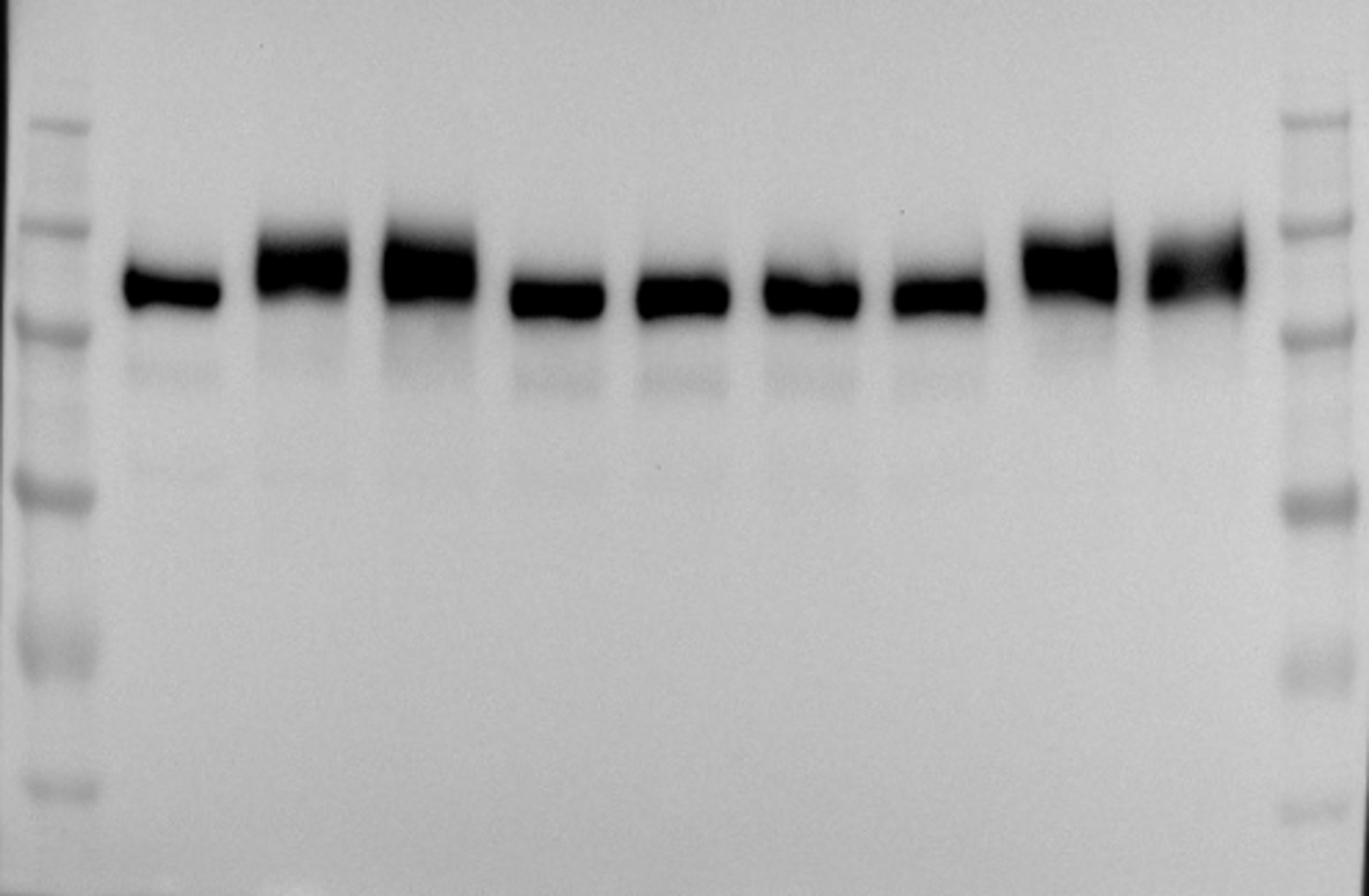

Supplement: Figure 2—figure supplement 2—source data 2. [file elife-88318-fig2-figsupp2-data2.zip › Fig2-Sup-HA input.tif]

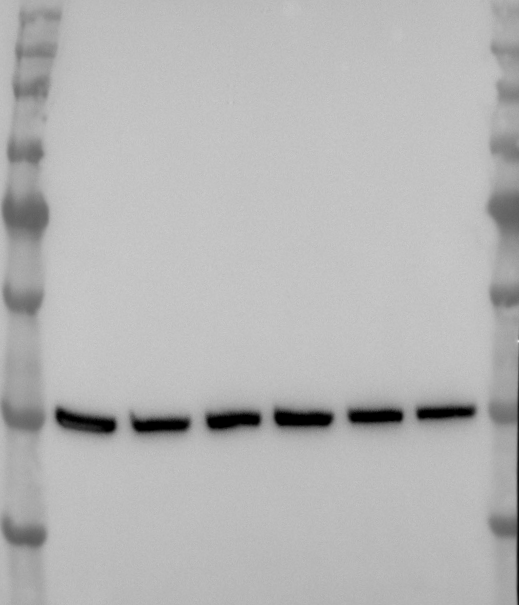

Supplement: Figure 3—source data 2. [file elife-88318-fig3-data2.zip › ACTIN-1.tif]

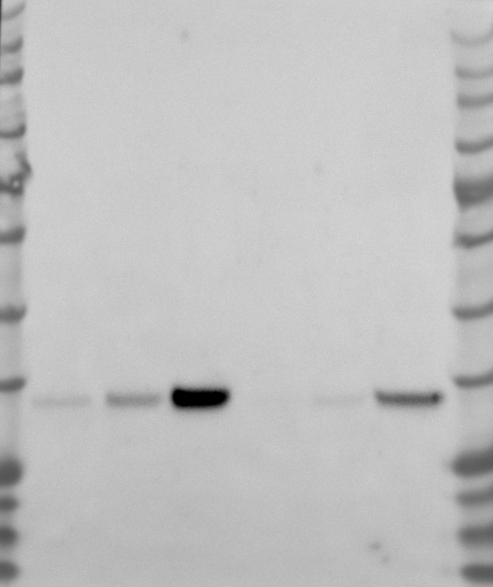

Supplement: Figure 3—source data 2. [file elife-88318-fig3-data2.zip › p-S6-1.tif]

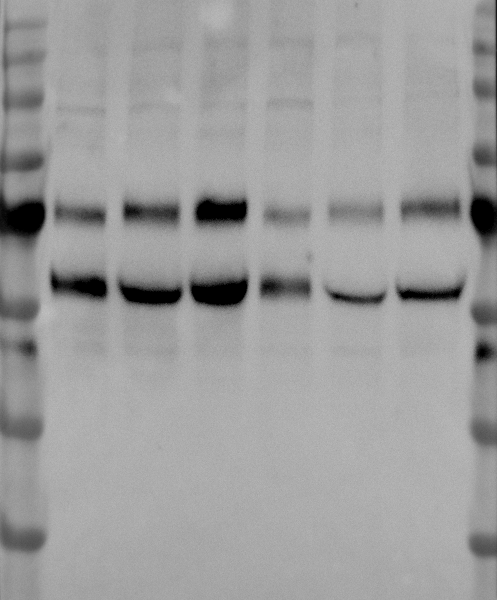

Supplement: Figure 3—source data 2. [file elife-88318-fig3-data2.zip › p-S6K-1.tif]

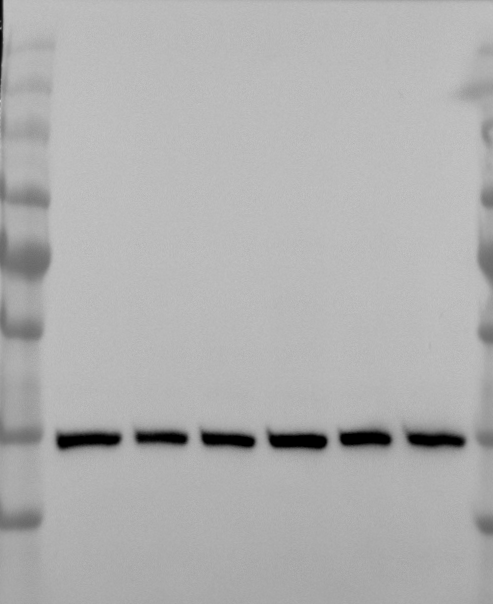

Supplement: Figure 3—source data 2. [file elife-88318-fig3-data2.zip › Repeat-ACTIN-2.tif]

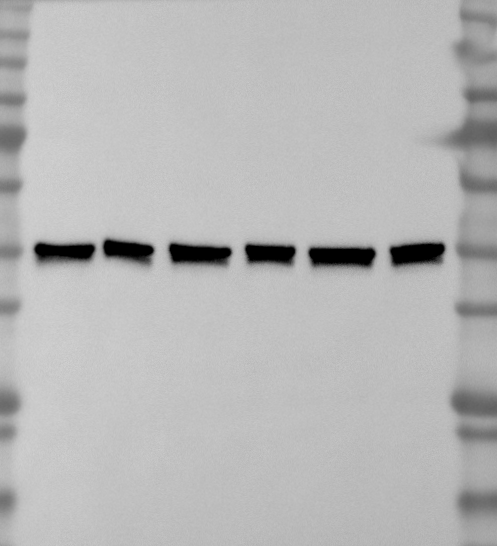

Supplement: Figure 3—source data 2. [file elife-88318-fig3-data2.zip › Repeat-ACTIN-3.tif]

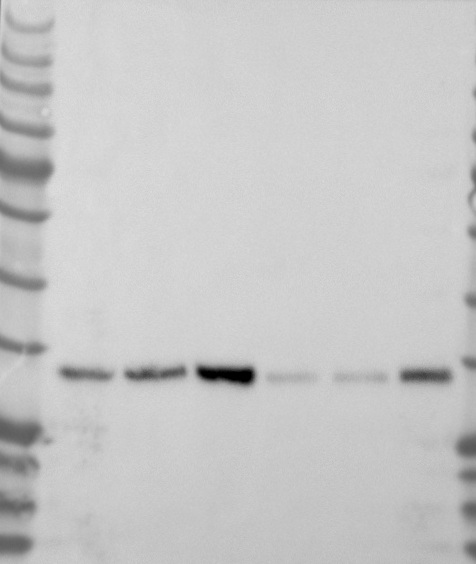

Supplement: Figure 3—source data 2. [file elife-88318-fig3-data2.zip › Repeat-p-S6-2.tif]

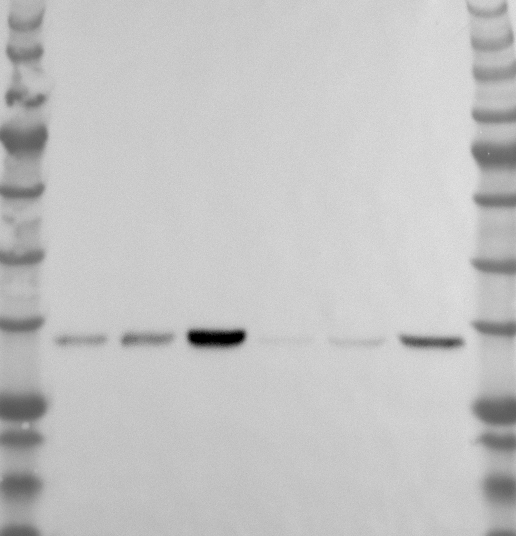

Supplement: Figure 3—source data 2. [file elife-88318-fig3-data2.zip › Repeat-p-S6-3.tif]

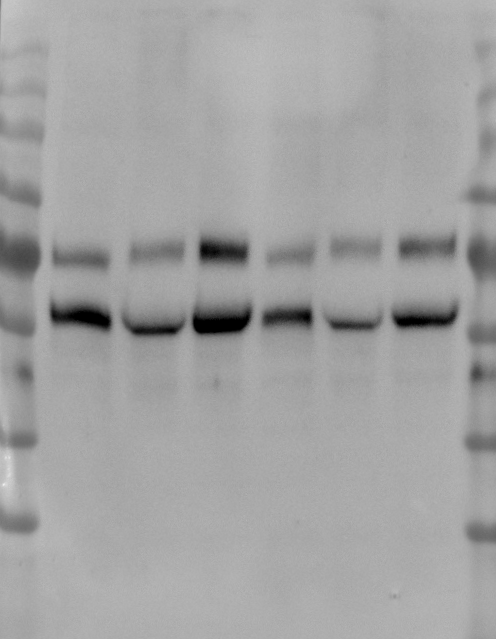

Supplement: Figure 3—source data 2. [file elife-88318-fig3-data2.zip › Repeat-p-S6K-2.tif]

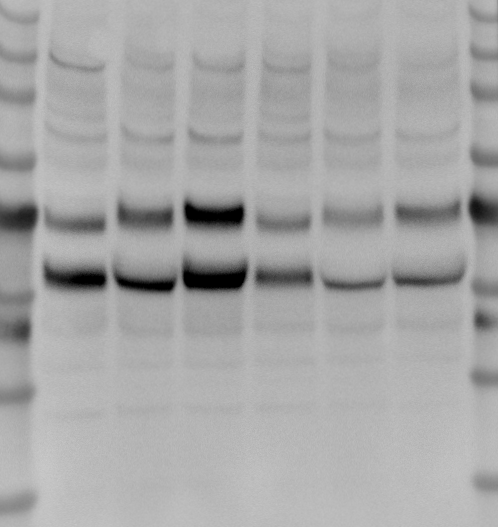

Supplement: Figure 3—source data 2. [file elife-88318-fig3-data2.zip › Repeat-p-S6K-3.tif]

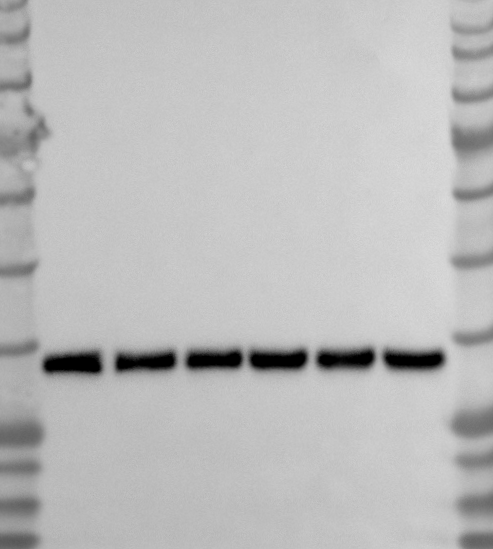

Supplement: Figure 3—source data 2. [file elife-88318-fig3-data2.zip › Repeat-S6-2.tif]

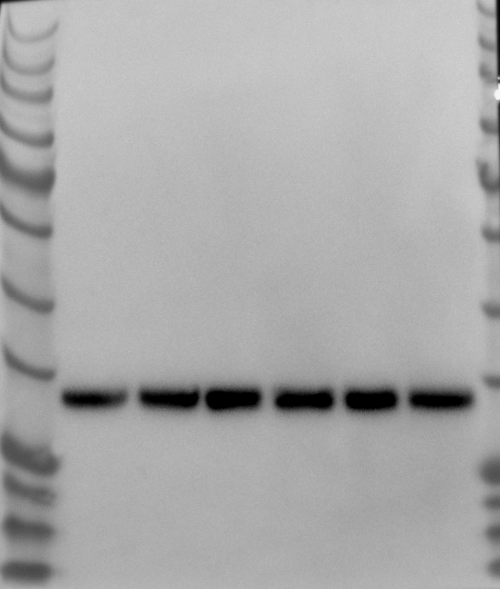

Supplement: Figure 3—source data 2. [file elife-88318-fig3-data2.zip › Repeat-S6-3.tif]

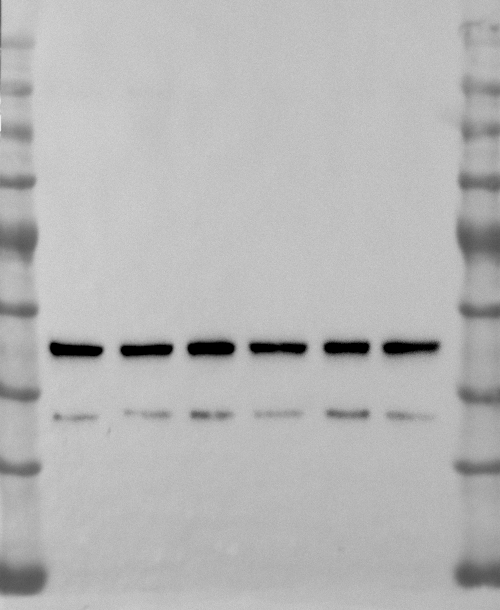

Supplement: Figure 3—source data 2. [file elife-88318-fig3-data2.zip › Repeat-S6K-2.tif]

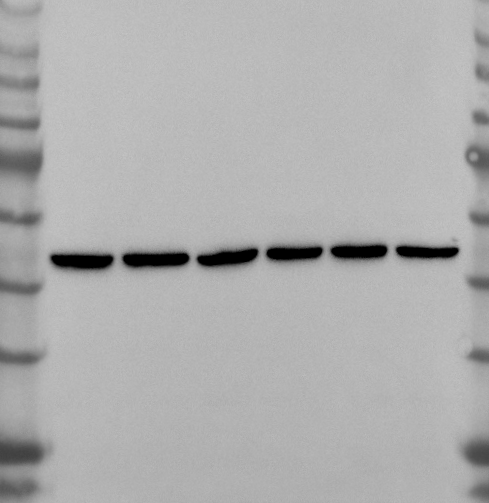

Supplement: Figure 3—source data 2. [file elife-88318-fig3-data2.zip › Repeat-S6K-3.tif]

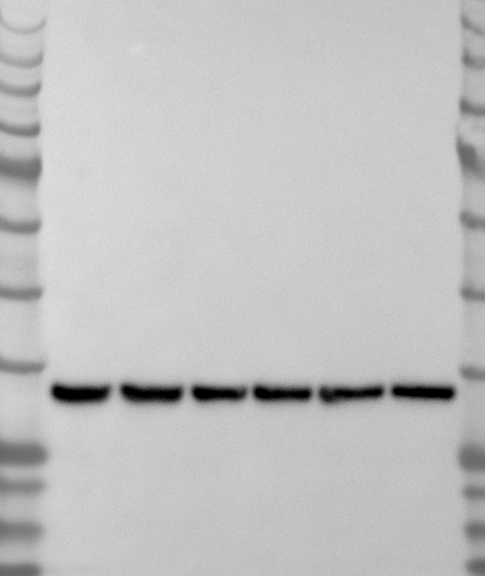

Supplement: Figure 3—source data 2. [file elife-88318-fig3-data2.zip › S6-1.tif]

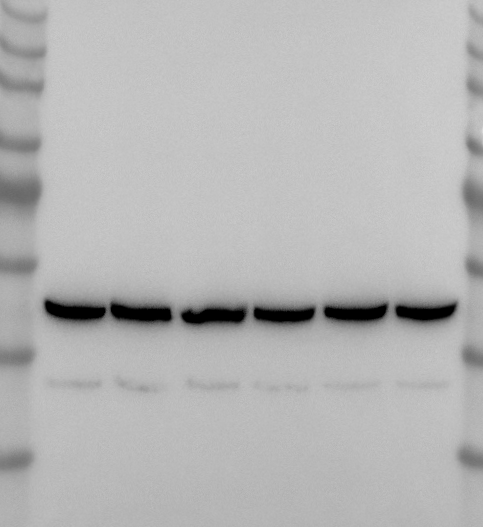

Supplement: Figure 3—source data 2. [file elife-88318-fig3-data2.zip › S6K-1.tif]

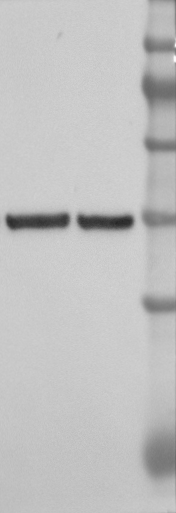

Supplement: Figure 3—source data 4. [file elife-88318-fig3-data4.zip › Actin.tif]

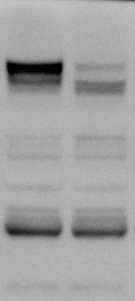

Supplement: Figure 3—source data 4. [file elife-88318-fig3-data4.zip › DYRK1A.tif]

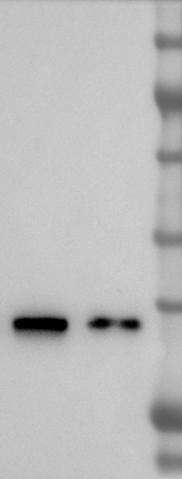

Supplement: Figure 3—source data 4. [file elife-88318-fig3-data4.zip › pS6.tif]

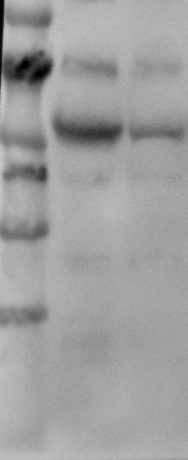

Supplement: Figure 3—source data 4. [file elife-88318-fig3-data4.zip › pS6K.tif]

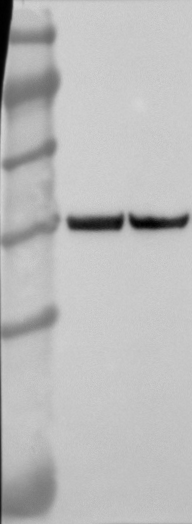

Supplement: Figure 3—source data 4. [file elife-88318-fig3-data4.zip › Repeat-Actin-1-full.tif]

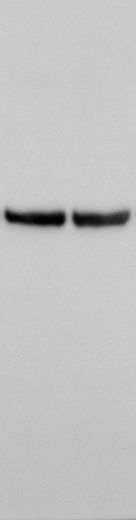

Supplement: Figure 3—source data 4. [file elife-88318-fig3-data4.zip › Repeat-Actin-2-full.tif]

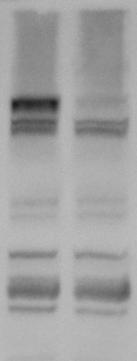

Supplement: Figure 3—source data 4. [file elife-88318-fig3-data4.zip › Repeat-DYRK1A-1-full.tif]

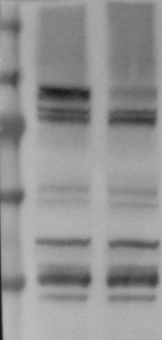

Supplement: Figure 3—source data 4. [file elife-88318-fig3-data4.zip › Repeat-DYRK1A-2-full.tif]

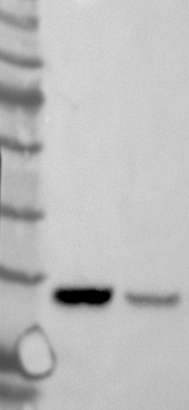

Supplement: Figure 3—source data 4. [file elife-88318-fig3-data4.zip › Repeat-pS6-1-full.tif]

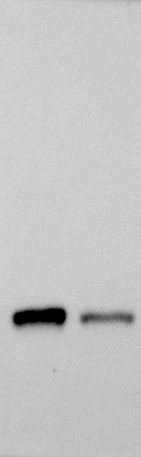

Supplement: Figure 3—source data 4. [file elife-88318-fig3-data4.zip › Repeat-pS6-2-full.tif]

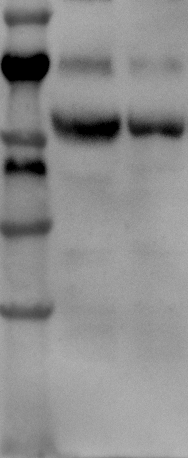

Supplement: Figure 3—source data 4. [file elife-88318-fig3-data4.zip › Repeat-pS6K-1-full.tif]

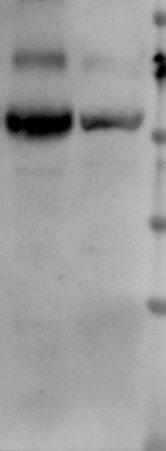

Supplement: Figure 3—source data 4. [file elife-88318-fig3-data4.zip › Repeat-pS6K-2-full.tif]

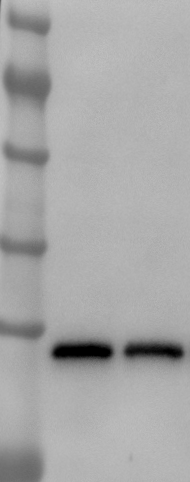

Supplement: Figure 3—source data 4. [file elife-88318-fig3-data4.zip › Repeat-S6-1-full.tif]

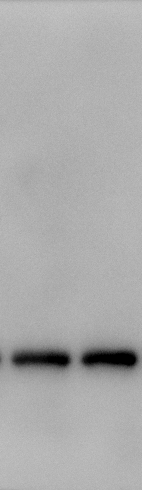

Supplement: Figure 3—source data 4. [file elife-88318-fig3-data4.zip › Repeat-S6-2-full.tif]

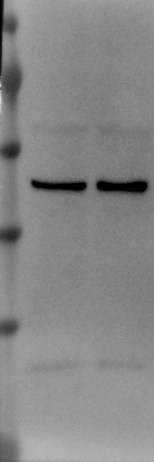

Supplement: Figure 3—source data 4. [file elife-88318-fig3-data4.zip › Repeat-S6K-1-full .tif]

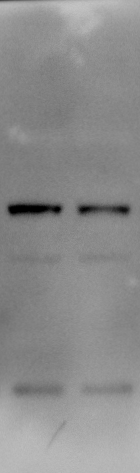

Supplement: Figure 3—source data 4. [file elife-88318-fig3-data4.zip › Repeat-S6K-2-full .tif]

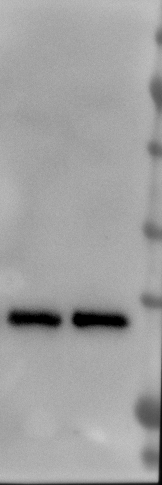

Supplement: Figure 3—source data 4. [file elife-88318-fig3-data4.zip › S6.tif]

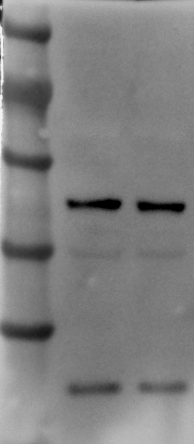

Supplement: Figure 3—source data 4. [file elife-88318-fig3-data4.zip › S6K.tif]

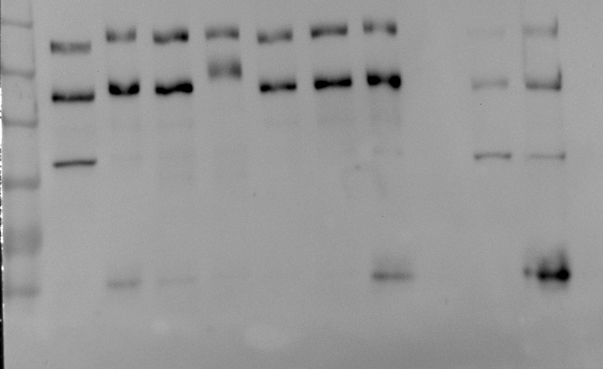

Supplement: Figure 4—source data 2. [file elife-88318-fig4-data2.zip › Figure 4A-TSC1-HA(lowerband).tif]

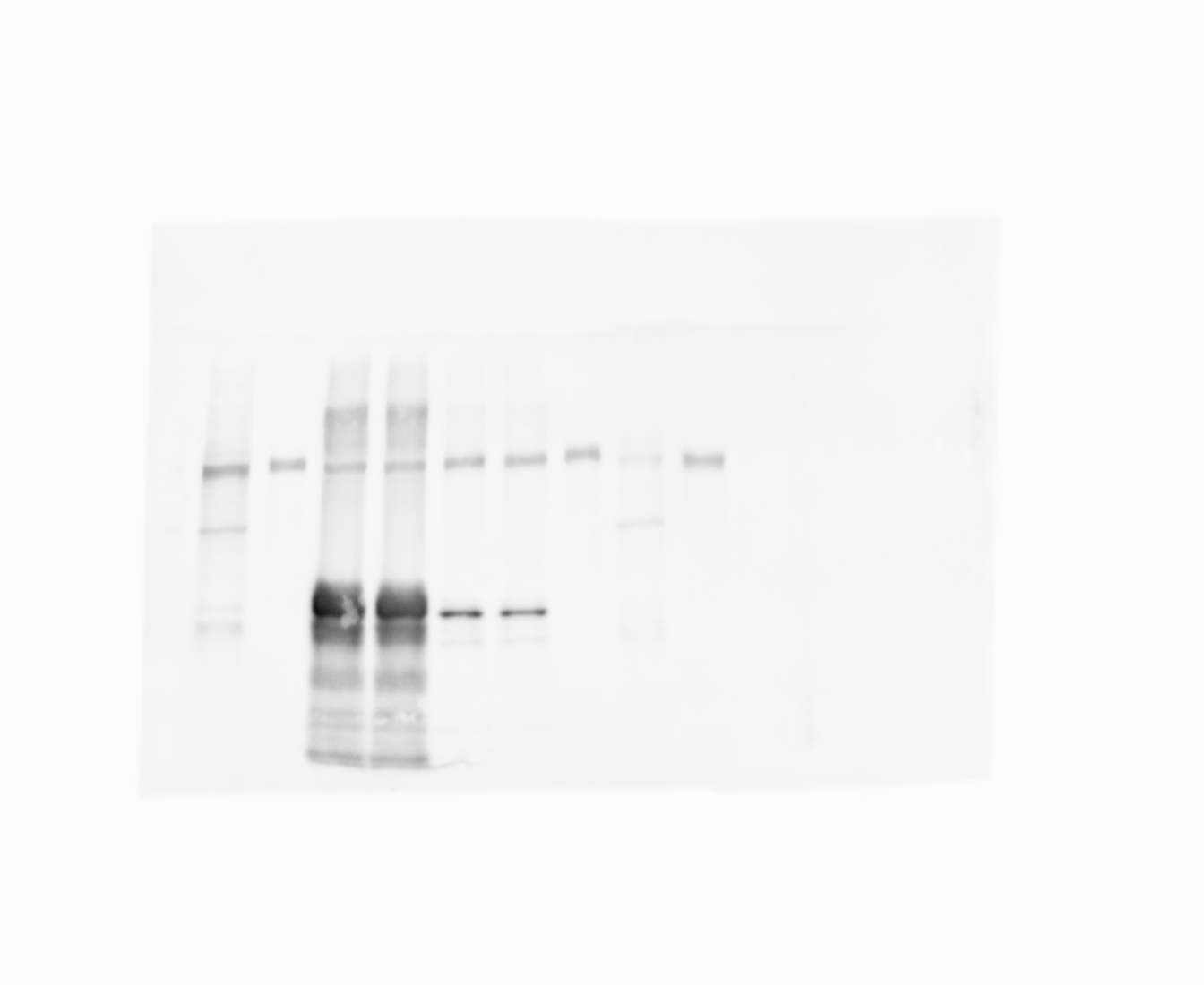

Supplement: Figure 4—source data 2. [file elife-88318-fig4-data2.zip › Figure 4A-DYRK1A.tif]

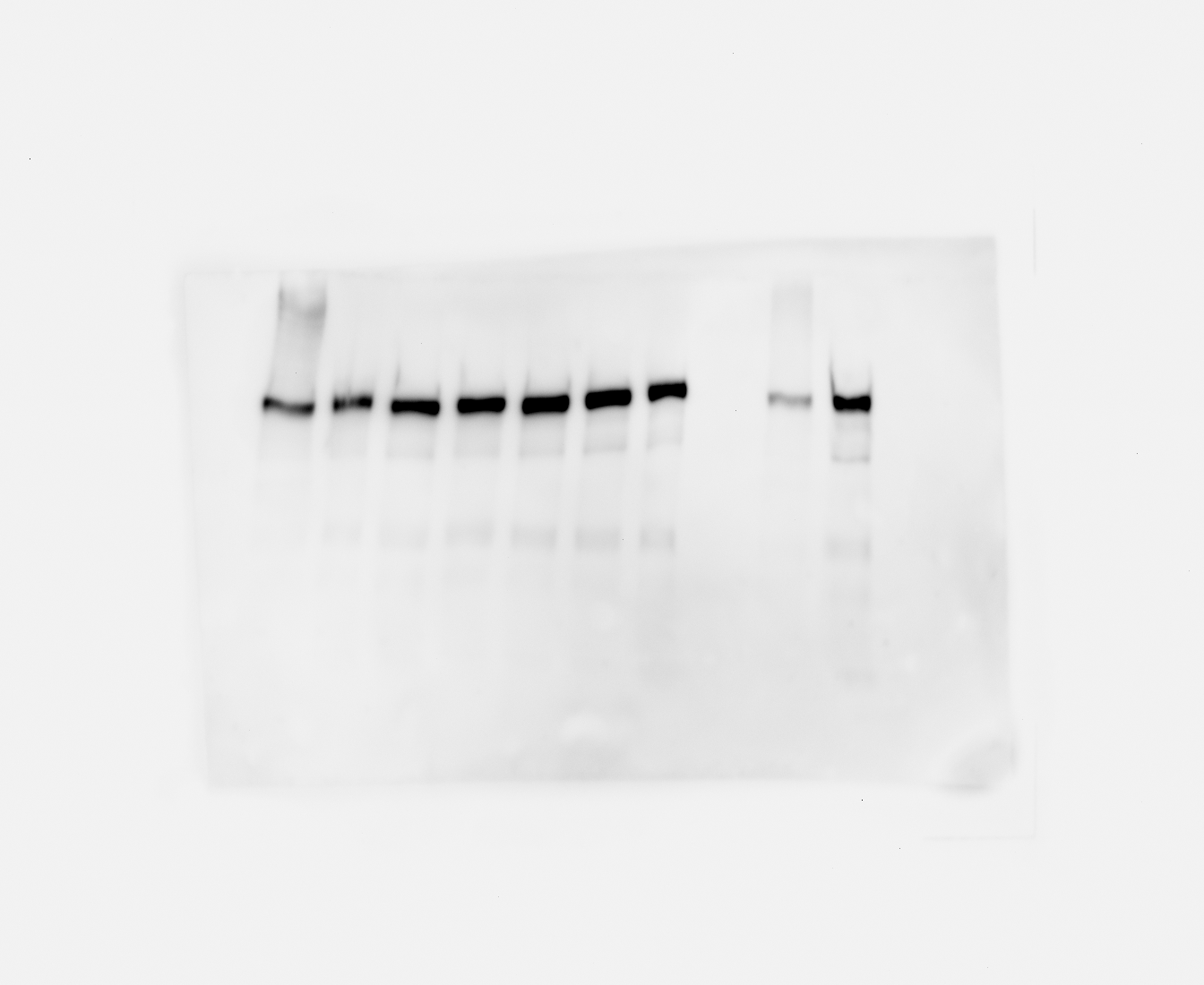

Supplement: Figure 4—source data 2. [file elife-88318-fig4-data2.zip › Figure 4A-pTSC2-1387.tif]

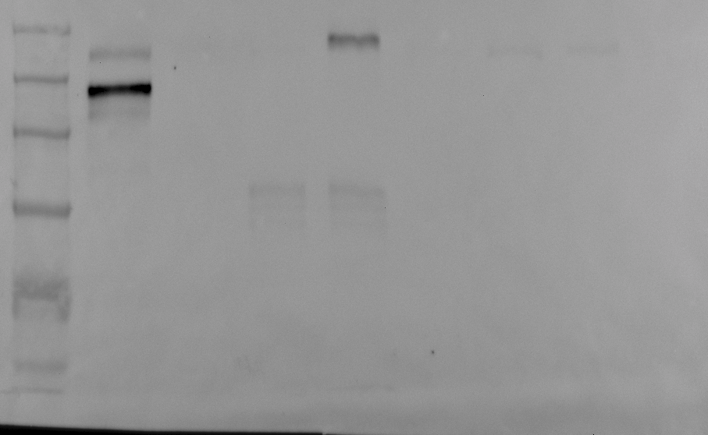

Supplement: Figure 4—source data 2. [file elife-88318-fig4-data2.zip › Figure 4A-pTSC2-1462.tif]

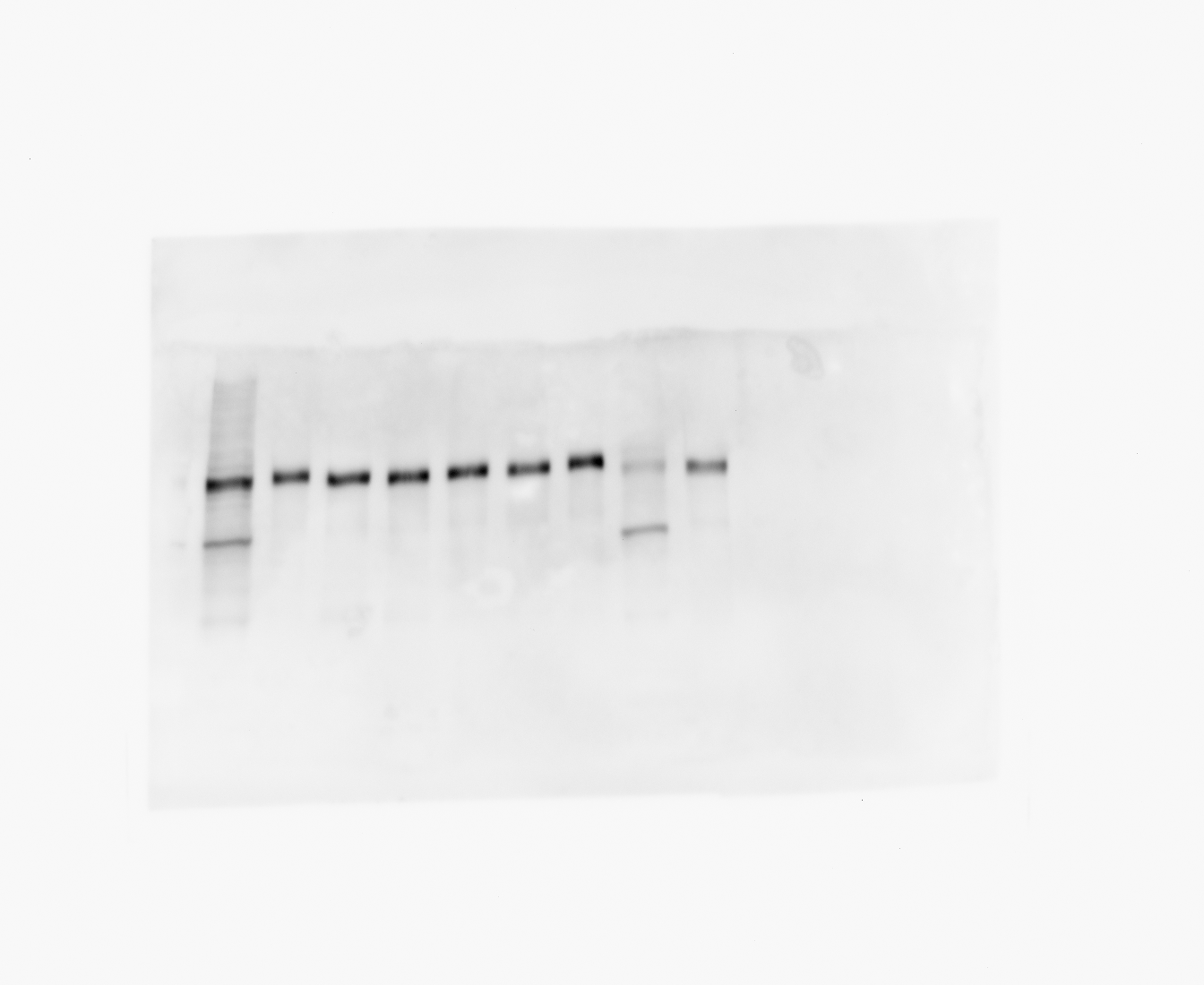

Supplement: Figure 4—source data 2. [file elife-88318-fig4-data2.zip › Figure 4A-pTSC2-939.tif]

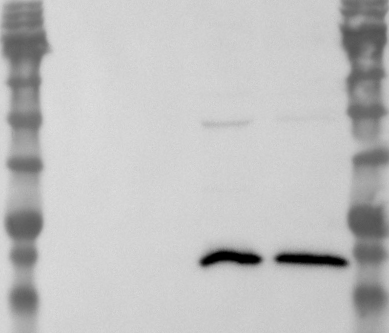

Supplement: Figure 4—source data 4. [file elife-88318-fig4-data4.zip › Flag(RHEB).tif]

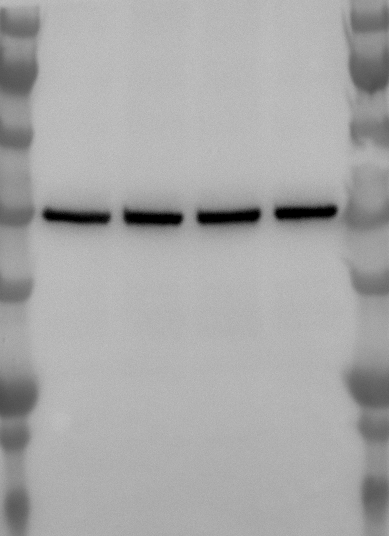

Supplement: Figure 4—source data 4. [file elife-88318-fig4-data4.zip › Actin.tif]

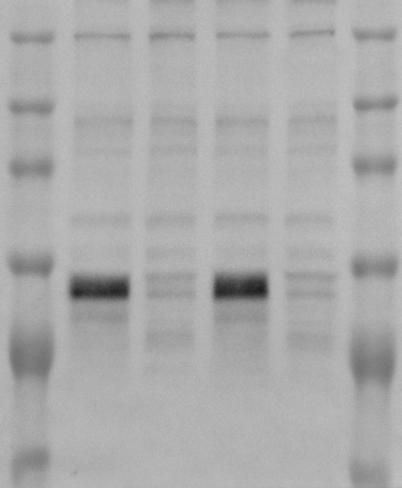

Supplement: Figure 4—source data 4. [file elife-88318-fig4-data4.zip › DYRK1A.tif]
