# Supplementary material for: DYRK1A interacts with the tuberous sclerosis complex and promotes mTORC1 activity
Source: eLife. 2024 Oct 22;12:RP88318. doi: 10.7554/eLife.88318 (PMC11495841; doi:10.7554/eLife.88318)
Supplement: Figure 1—source data 3. [file elife-88318-fig1-data3.zip › Figure 1C-source data.pptx]

## Slide 1
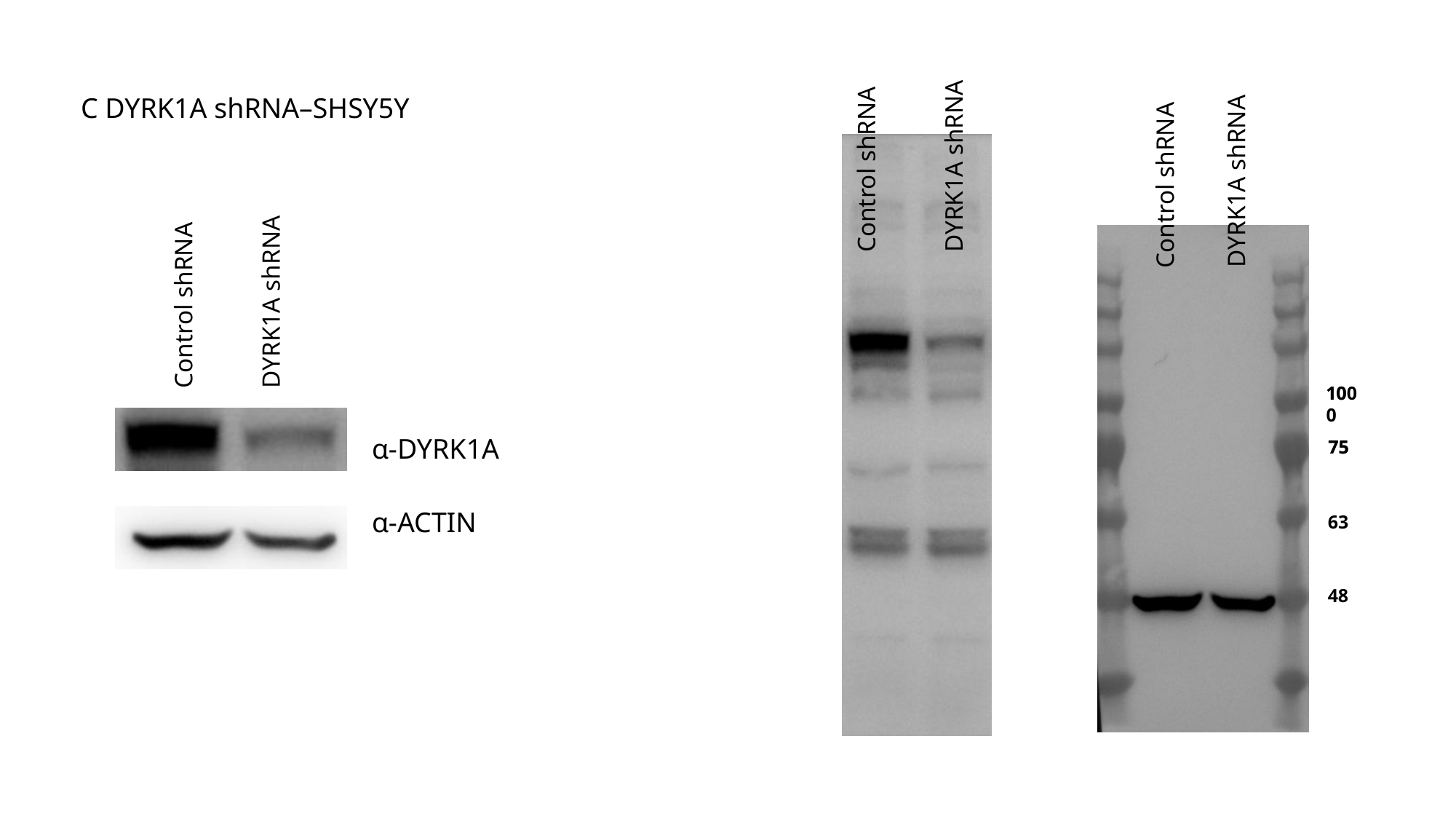

C DYRK1A shRNA–SHSY5Y
Control shRNA
DYRK1A shRNA
Control shRNA
DYRK1A shRNA
Control shRNA
DYRK1A shRNA
100
100
α-DYRK1A
75
75
α-ACTIN
63
48
