# Supplementary material for: DYRK1A interacts with the tuberous sclerosis complex and promotes mTORC1 activity
Source: eLife. 2024 Oct 22;12:RP88318. doi: 10.7554/eLife.88318 (PMC11495841; doi:10.7554/eLife.88318)
Supplement: Figure 1—source data 5. [file elife-88318-fig1-data5.zip › Figure 1E-source data.pptx]

## Slide 1
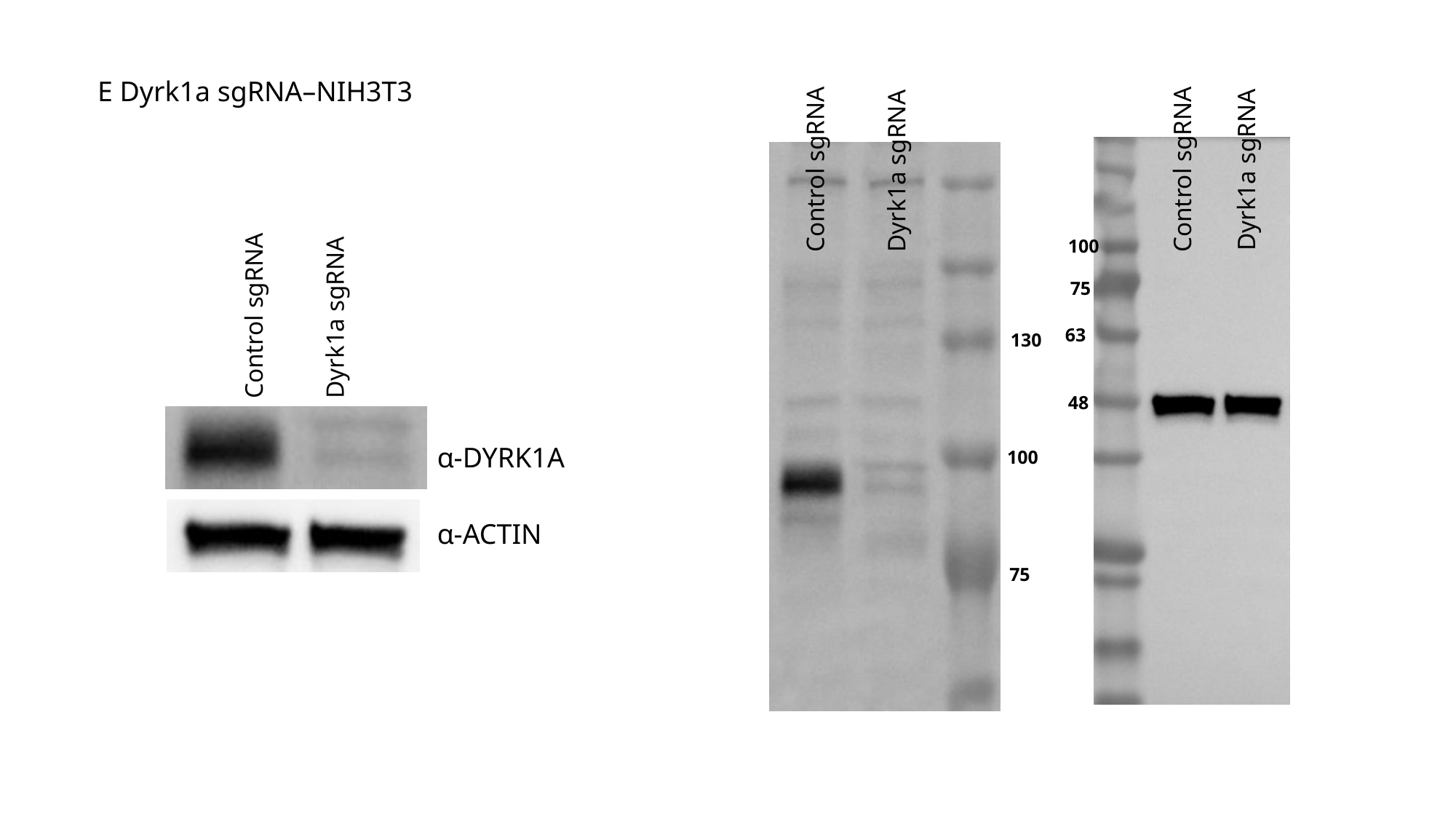

E Dyrk1a sgRNA–NIH3T3
Dyrk1a sgRNA
Control sgRNA
Control sgRNA
Dyrk1a sgRNA
100
75
Control sgRNA
Dyrk1a sgRNA
63
130
48
α-DYRK1A
100
α-ACTIN
75
