# Supplementary material for: DYRK1A interacts with the tuberous sclerosis complex and promotes mTORC1 activity
Source: eLife. 2024 Oct 22;12:RP88318. doi: 10.7554/eLife.88318 (PMC11495841; doi:10.7554/eLife.88318)
Supplement: Figure 2—source data 1. [file elife-88318-fig2-data1.zip › Figure 2B-source data.pptx]

## Slide 1
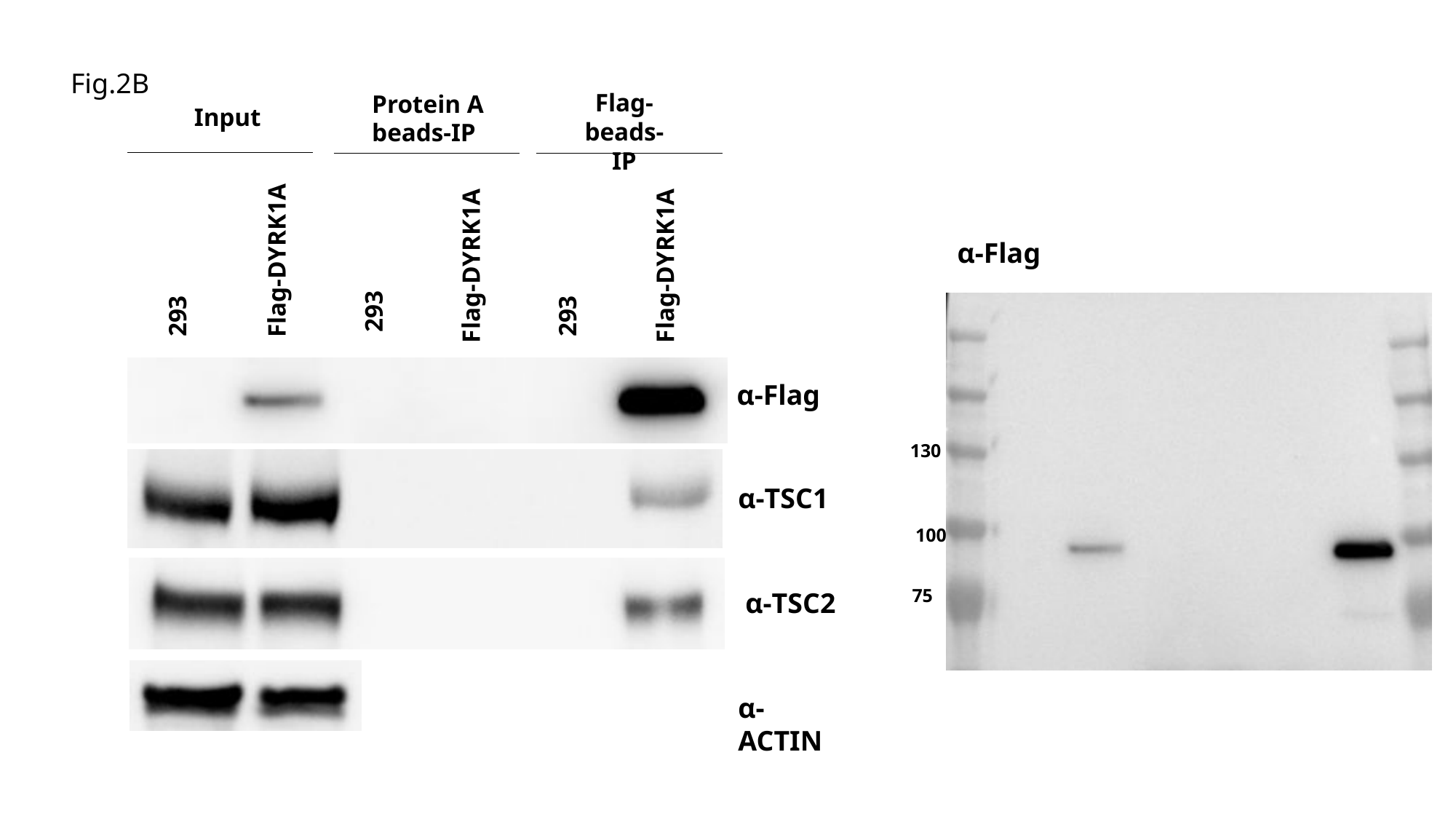

Fig.2B
Flag-
beads-IP
Protein A
beads-IP
Input
Flag-DYRK1A
Flag-DYRK1A
Flag-DYRK1A
293
293
293
α-Flag
α-TSC1
α-TSC2
α-ACTIN
α-Flag
130
100
75

## Slide 2
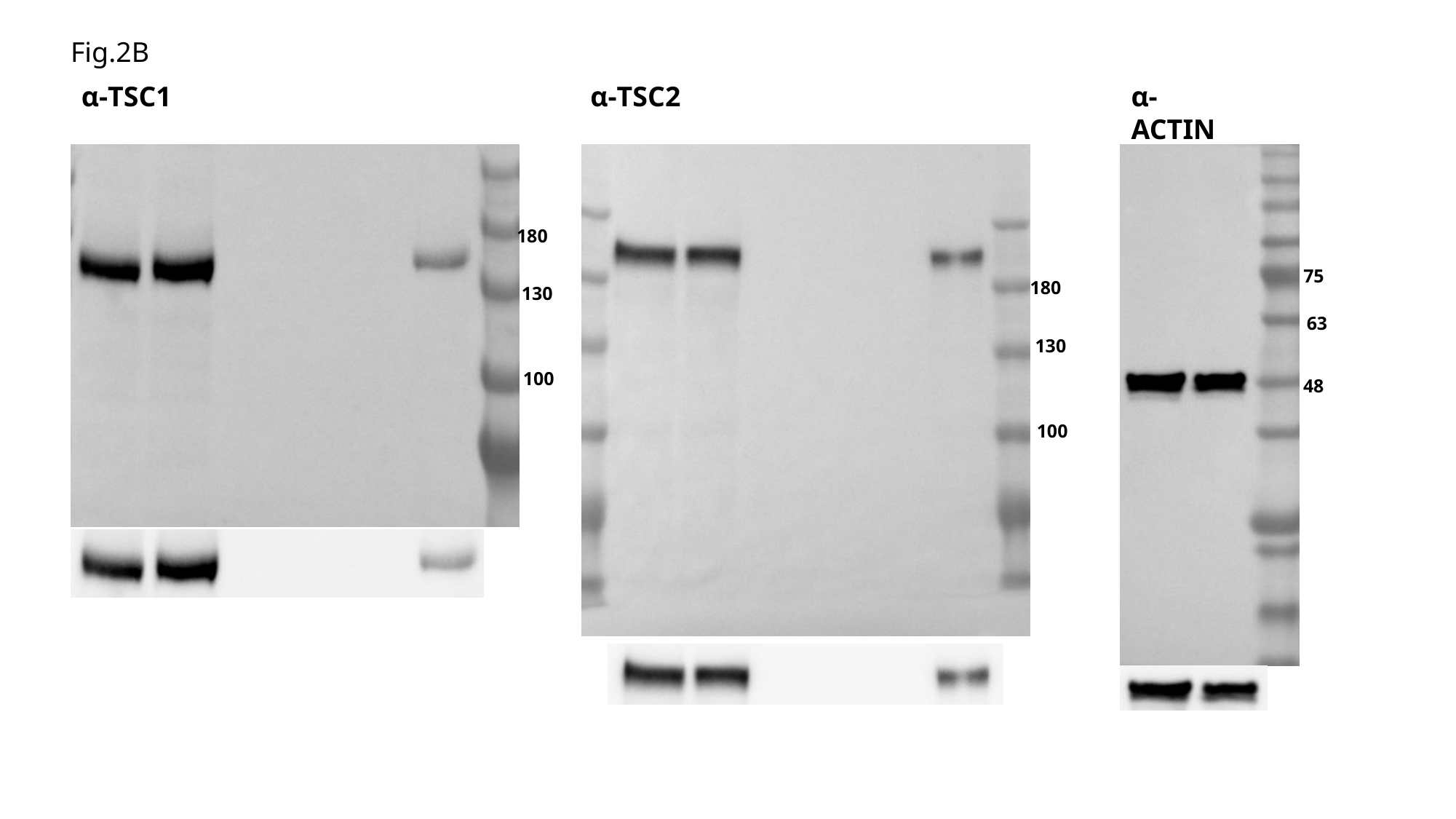

Fig.2B
α-TSC1
α-TSC2
α-ACTIN
180
75
180
130
63
130
100
48
100
