# Supplementary material for: DYRK1A interacts with the tuberous sclerosis complex and promotes mTORC1 activity
Source: eLife. 2024 Oct 22;12:RP88318. doi: 10.7554/eLife.88318 (PMC11495841; doi:10.7554/eLife.88318)
Supplement: Figure 2—source data 5. [file elife-88318-fig2-data5.zip › Figure 2D-source data.pptx]

## Slide 1
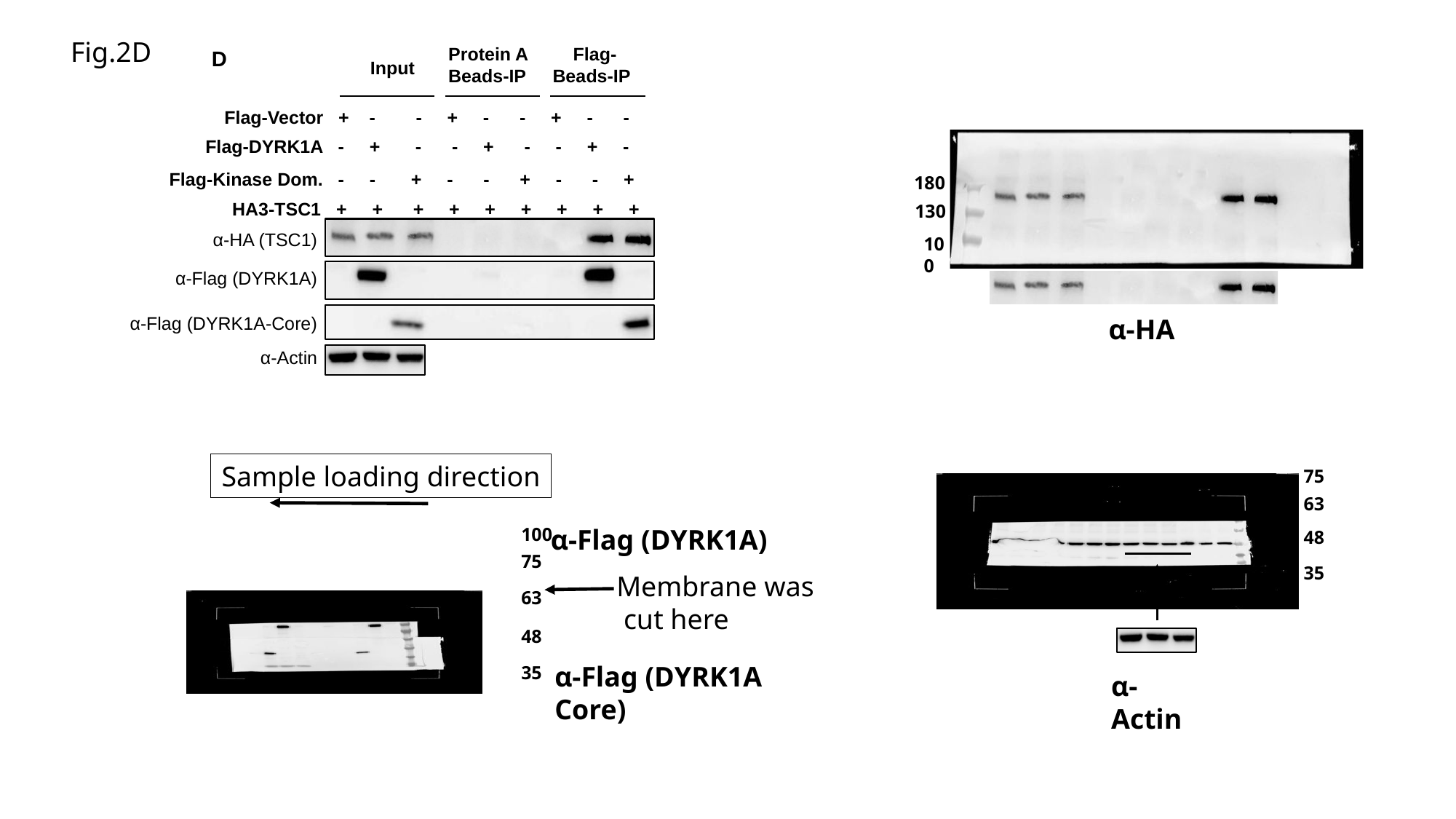

Fig.2D
Protein A
Beads-IP
 Flag-
Beads-IP
D
Input
Flag-Vector + - - + - - + - -
Flag-DYRK1A - + - - + - - + -
Flag-Kinase Dom. - - + - - + - - +
HA3-TSC1 + + + + + + + + +
α-HA (TSC1)
α-Flag (DYRK1A)
α-Flag (DYRK1A-Core)
α-Actin
180
130
100
α-HA
Sample loading direction
α-Flag (DYRK1A)
100
75
63
48
α-Flag (DYRK1A Core)
35
Membrane was
 cut here
75
63
48
35
α-Actin
