# Supplementary material for: DYRK1A interacts with the tuberous sclerosis complex and promotes mTORC1 activity
Source: eLife. 2024 Oct 22;12:RP88318. doi: 10.7554/eLife.88318 (PMC11495841; doi:10.7554/eLife.88318)
Supplement: Figure 2—figure supplement 1—source data 1. [file elife-88318-fig2-figsupp1-data1.zip › Figure 2-figure supplement 1-source data.pptx]

## Slide 1
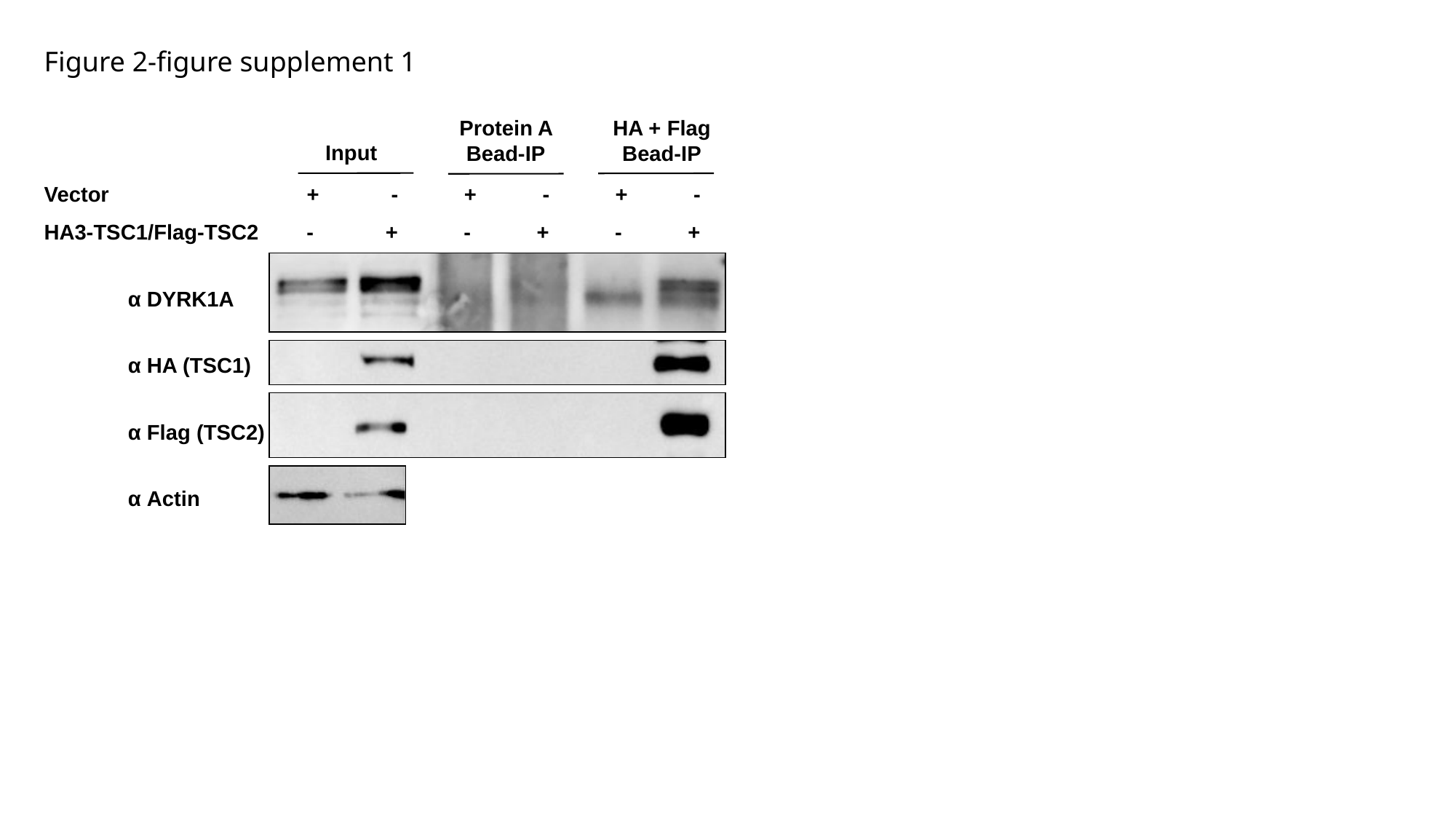

Figure 2-figure supplement 1
Protein A Bead-IP
HA + Flag Bead-IP
Input
Vector + - + - + -
HA3-TSC1/Flag-TSC2 - + - + - +
α DYRK1A
α HA (TSC1)
α Flag (TSC2)
α Actin

## Slide 2
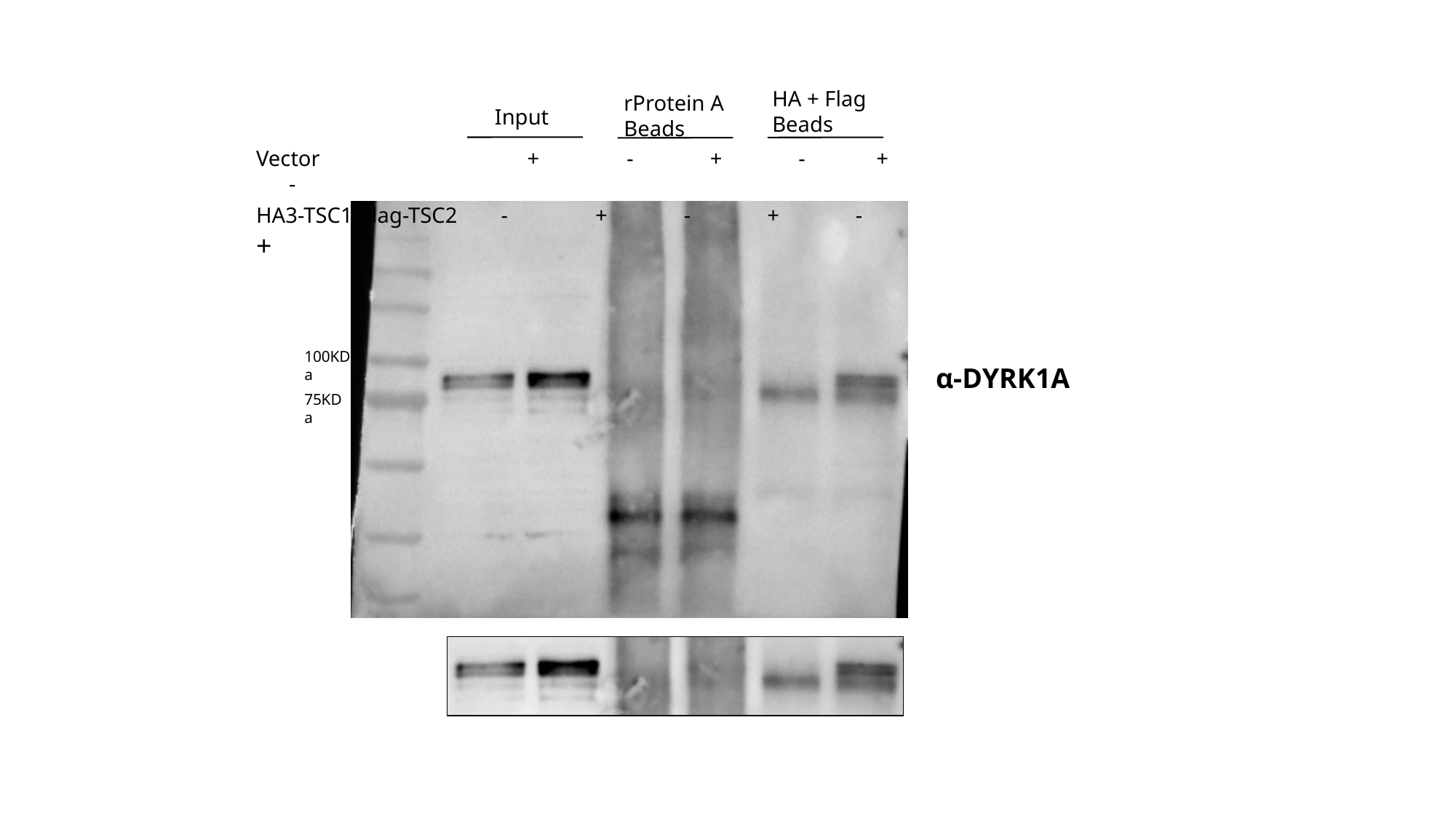

HA + Flag Beads
rProtein A Beads
Input
Vector + - + - + -
HA3-TSC1/Flag-TSC2 - + - + - +
100KDa
α-DYRK1A
75KDa

## Slide 3
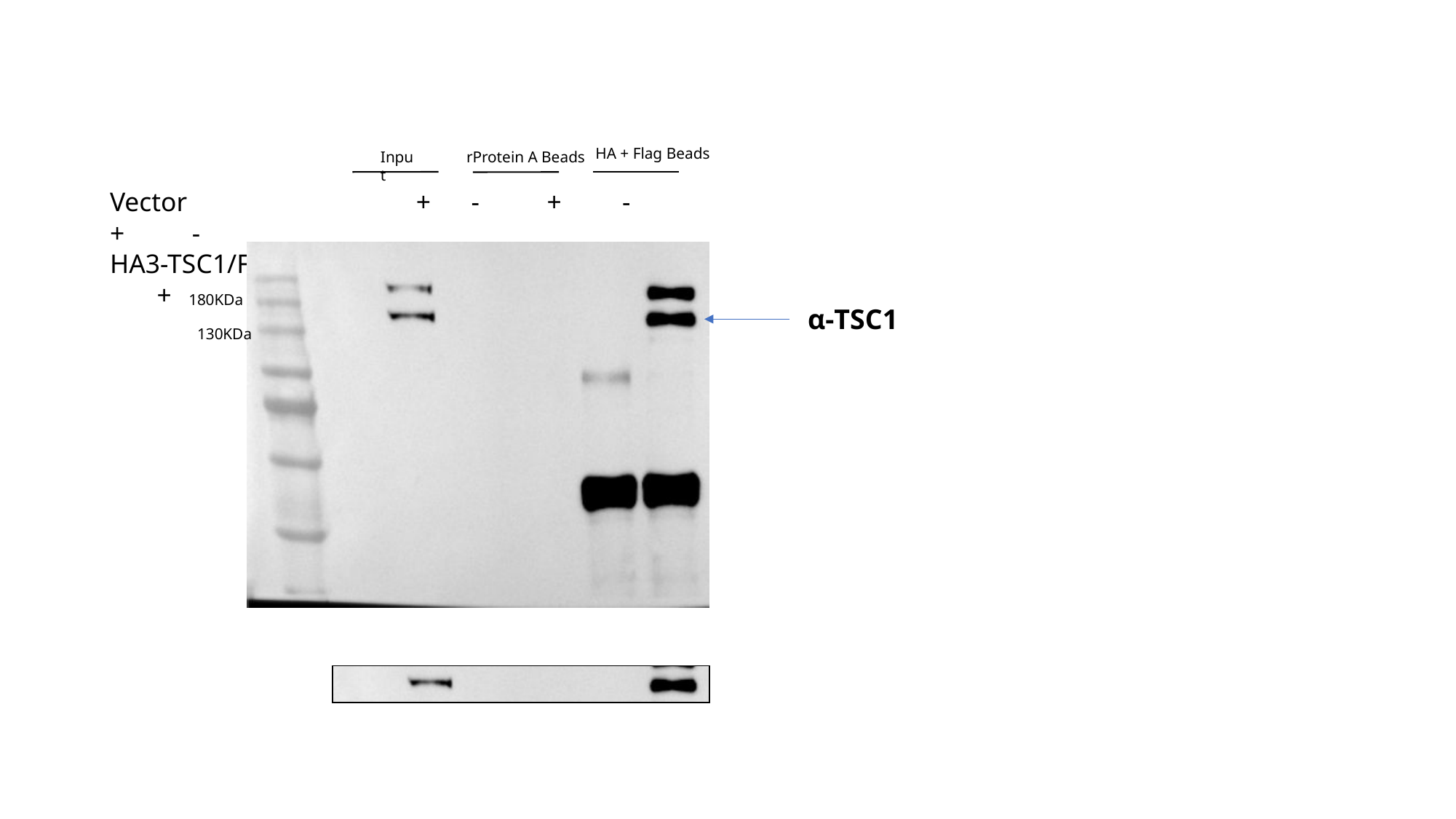

HA + Flag Beads
Input
rProtein A Beads
Vector + - + - + -
HA3-TSC1/Flag-TSC2 - + - + - +
180KDa
α-TSC1
130KDa

## Slide 4
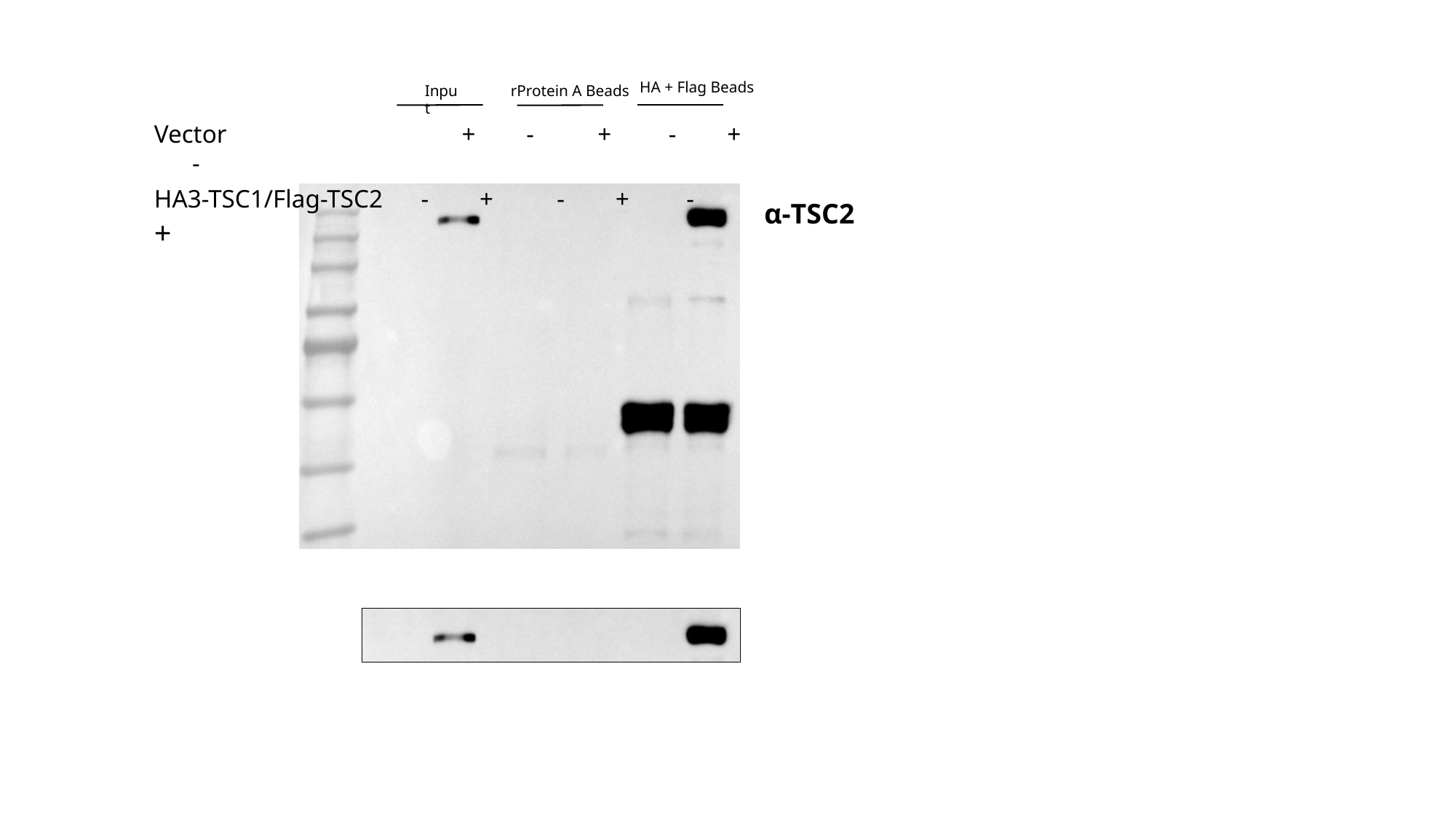

HA + Flag Beads
Input
rProtein A Beads
Vector + - + - + -
HA3-TSC1/Flag-TSC2 - + - + - +
α-TSC2

## Slide 5
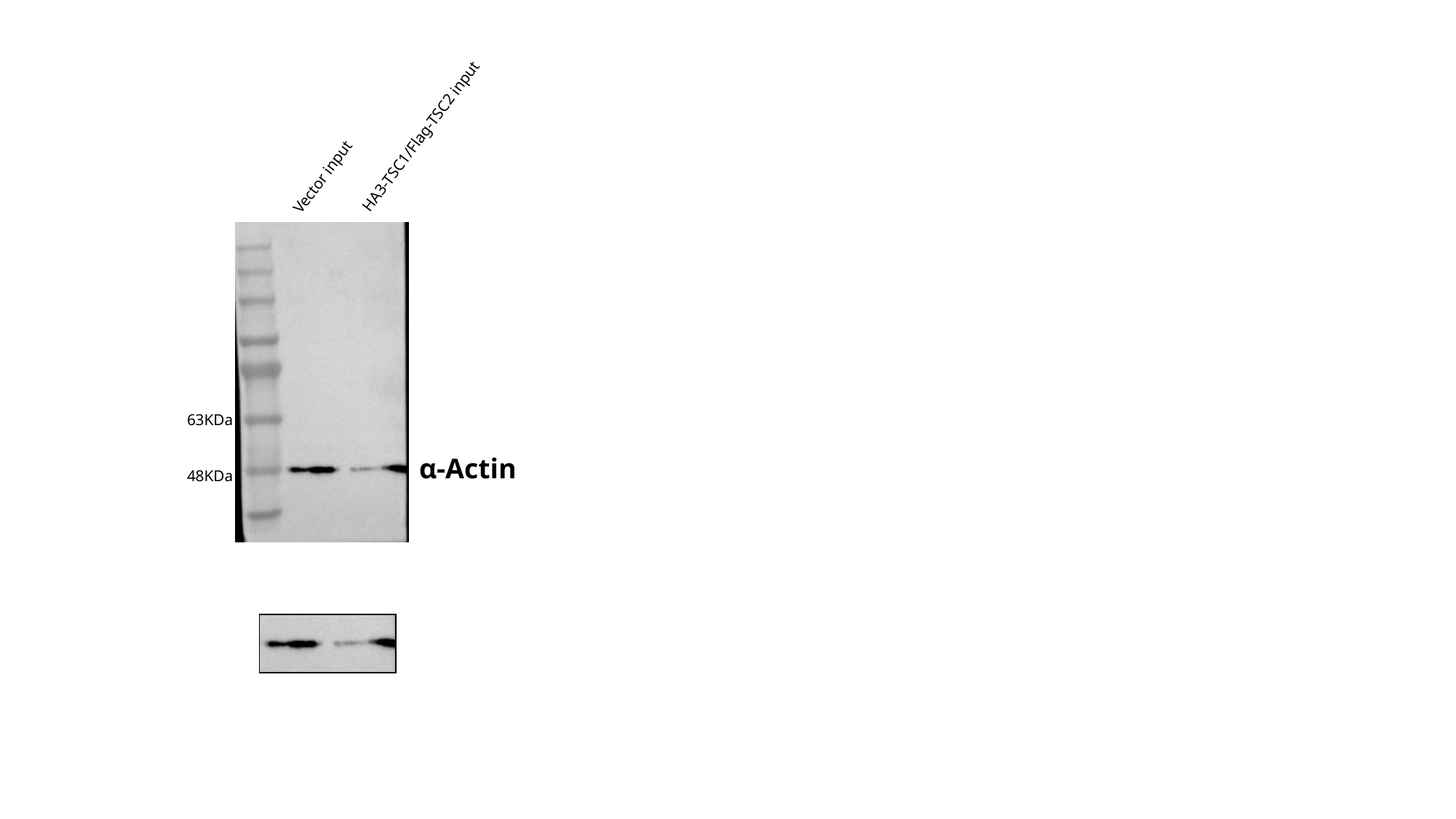

HA3-TSC1/Flag-TSC2 input
Vector input
63KDa
α-Actin
48KDa
