# Supplementary material for: DYRK1A interacts with the tuberous sclerosis complex and promotes mTORC1 activity
Source: eLife. 2024 Oct 22;12:RP88318. doi: 10.7554/eLife.88318 (PMC11495841; doi:10.7554/eLife.88318)
Supplement: Figure 2—figure supplement 2—source data 1. [file elife-88318-fig2-figsupp2-data1.zip › Figure 2-figure supplement 2-source data.pptx]

## Slide 1
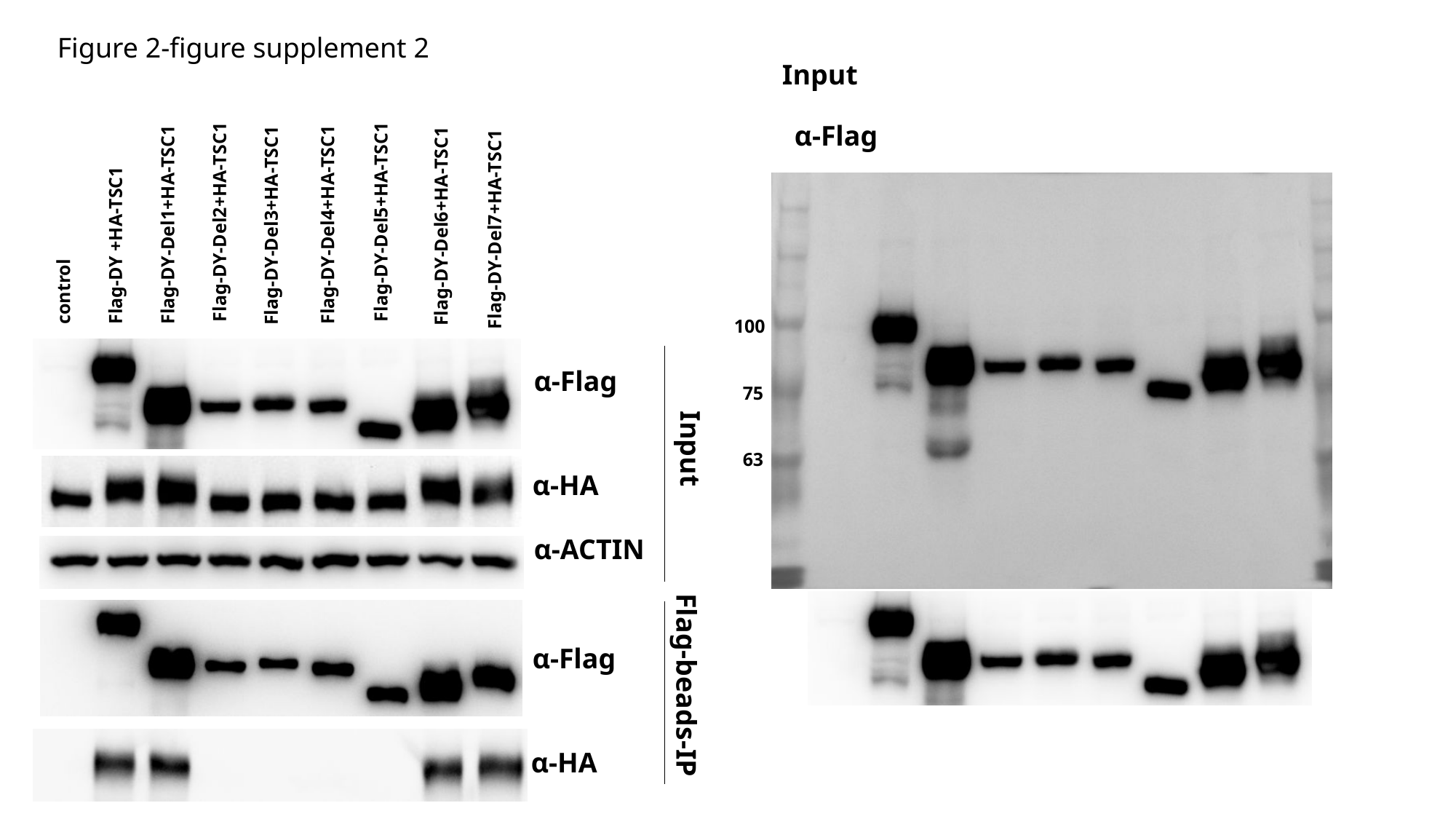

Figure 2-figure supplement 2
Input
α-Flag
Flag-DY-Del5+HA-TSC1
Flag-DY-Del2+HA-TSC1
Flag-DY-Del4+HA-TSC1
Flag-DY-Del1+HA-TSC1
Flag-DY-Del3+HA-TSC1
Flag-DY-Del6+HA-TSC1
Flag-DY-Del7+HA-TSC1
Flag-DY +HA-TSC1
control
100
α-Flag
75
Input
63
α-HA
α-ACTIN
α-Flag
Flag-beads-IP
α-HA

## Slide 2
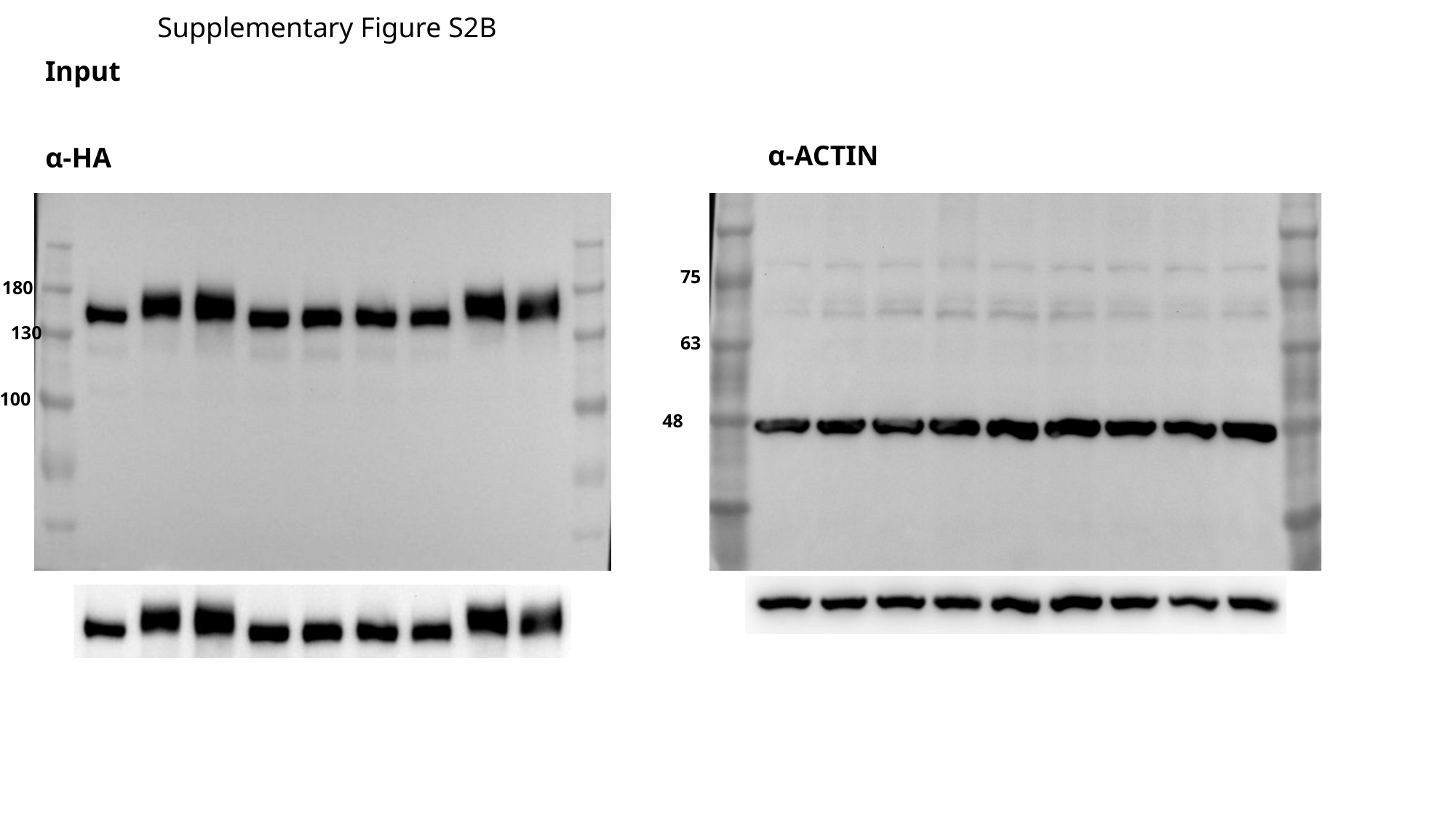

Supplementary Figure S2B
Input
α-ACTIN
α-HA
75
180
130
63
100
48

## Slide 3
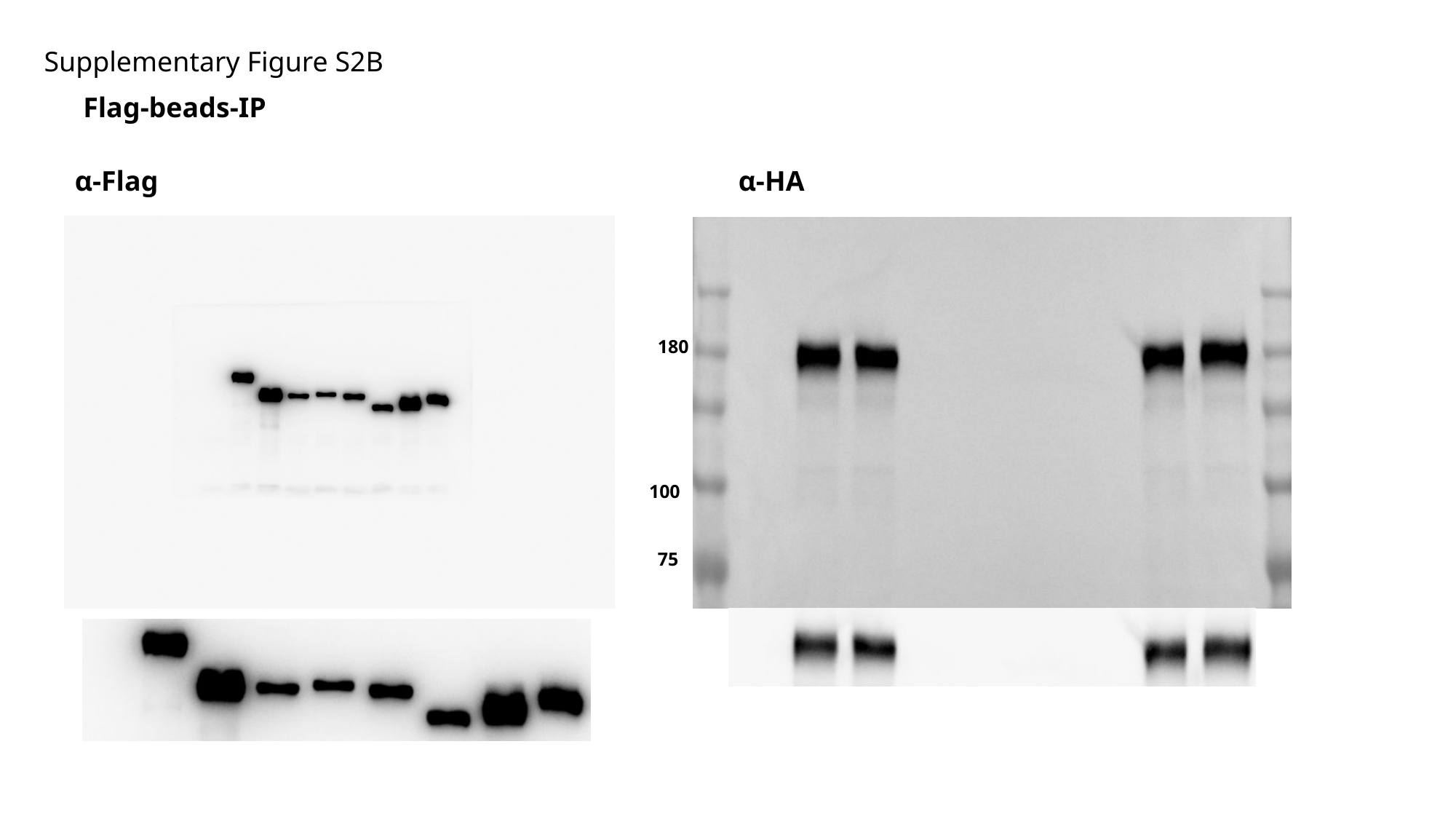

Supplementary Figure S2B
Flag-beads-IP
α-Flag
α-HA
180
100
75
