# Supplementary material for: DYRK1A interacts with the tuberous sclerosis complex and promotes mTORC1 activity
Source: eLife. 2024 Oct 22;12:RP88318. doi: 10.7554/eLife.88318 (PMC11495841; doi:10.7554/eLife.88318)
Supplement: Figure 3—source data 1. [file elife-88318-fig3-data1.zip › Figure 3A-source data.pptx]

## Slide 1
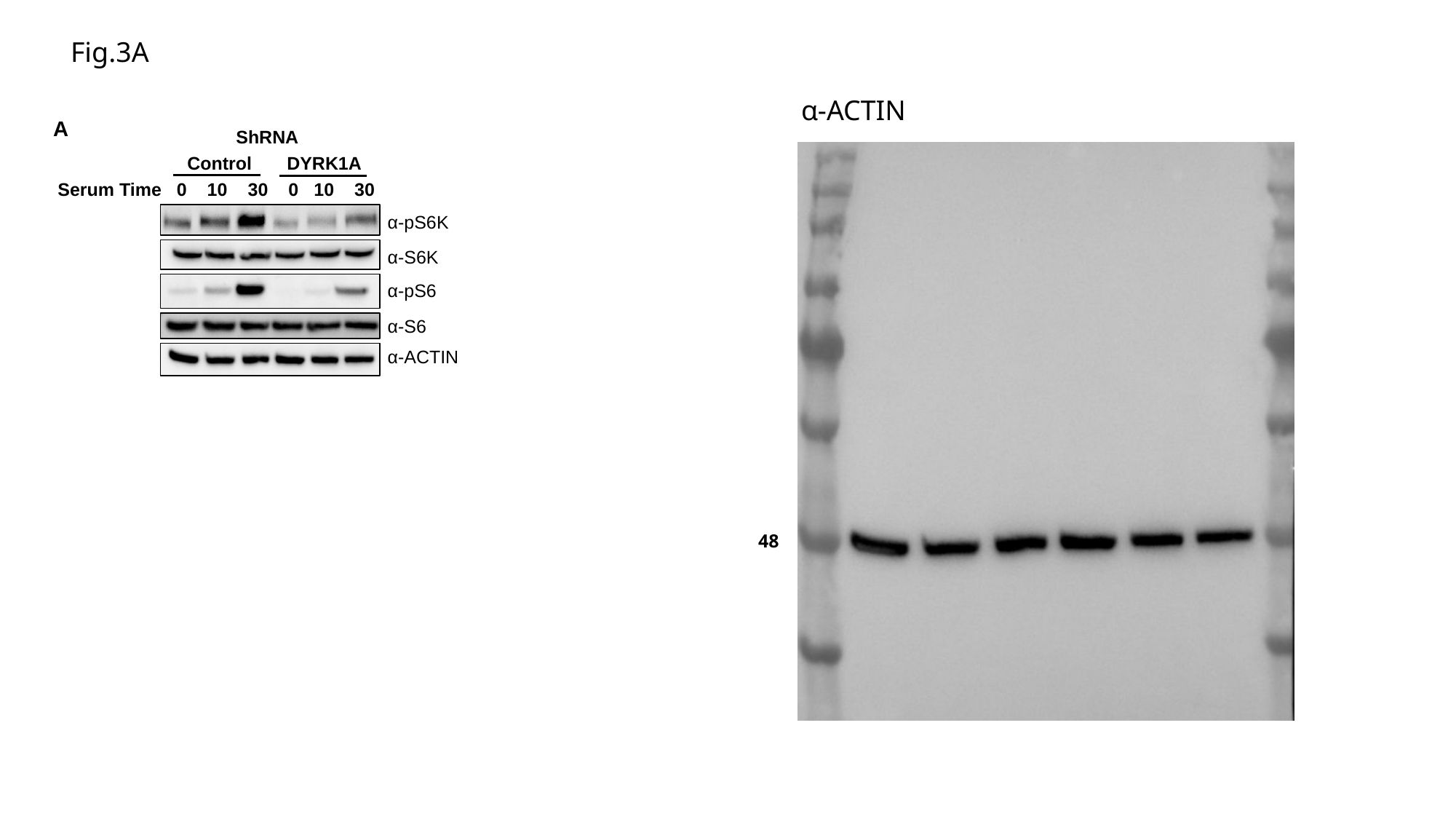

Fig.3A
α-ACTIN
A
ShRNA
Control
DYRK1A
Serum Time 0 10 30 0 10 30
α-pS6K
α-S6K
α-pS6
α-S6
α-ACTIN
48

## Slide 2
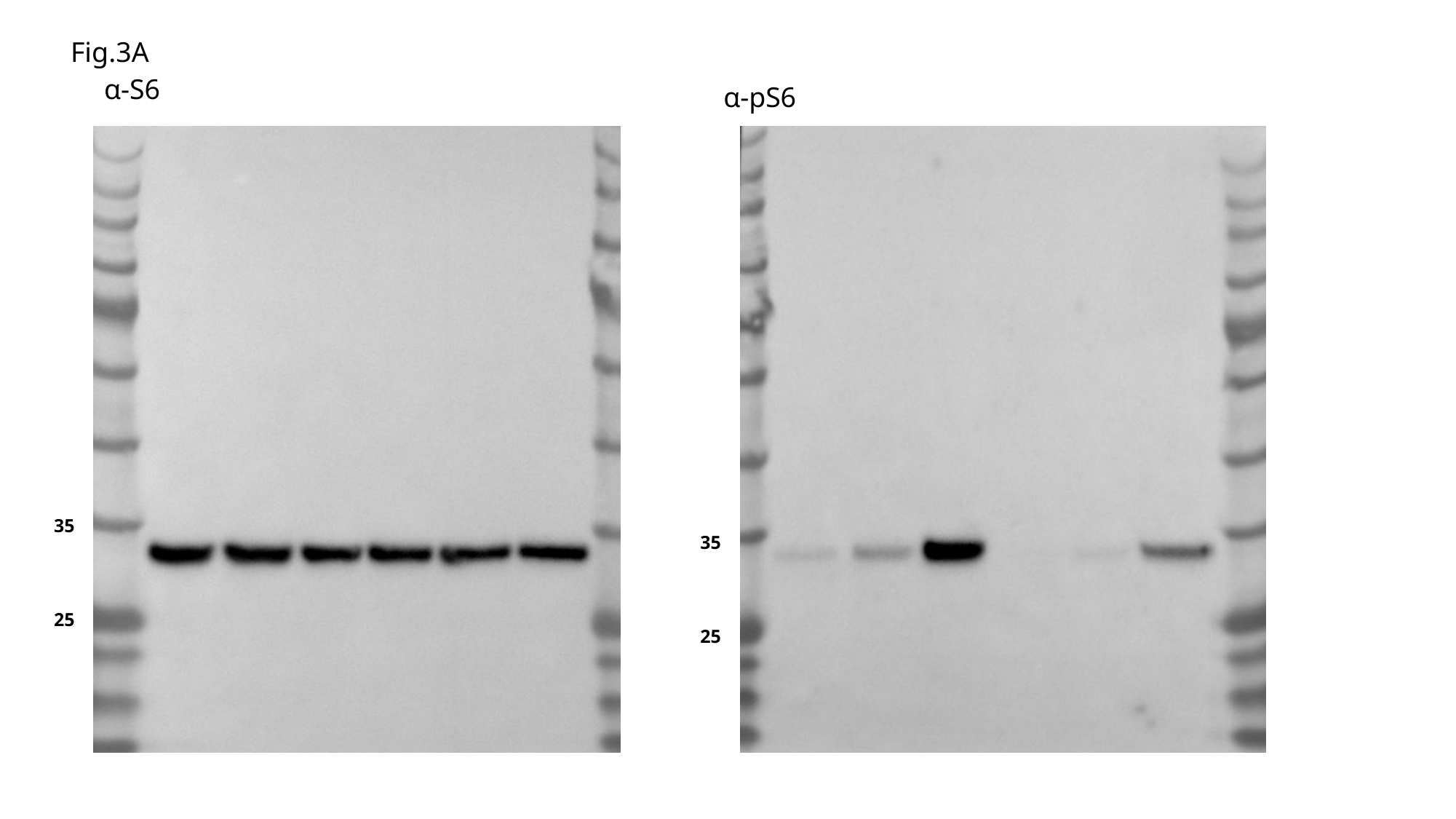

Fig.3A
α-S6
α-pS6
35
35
25
25

## Slide 3
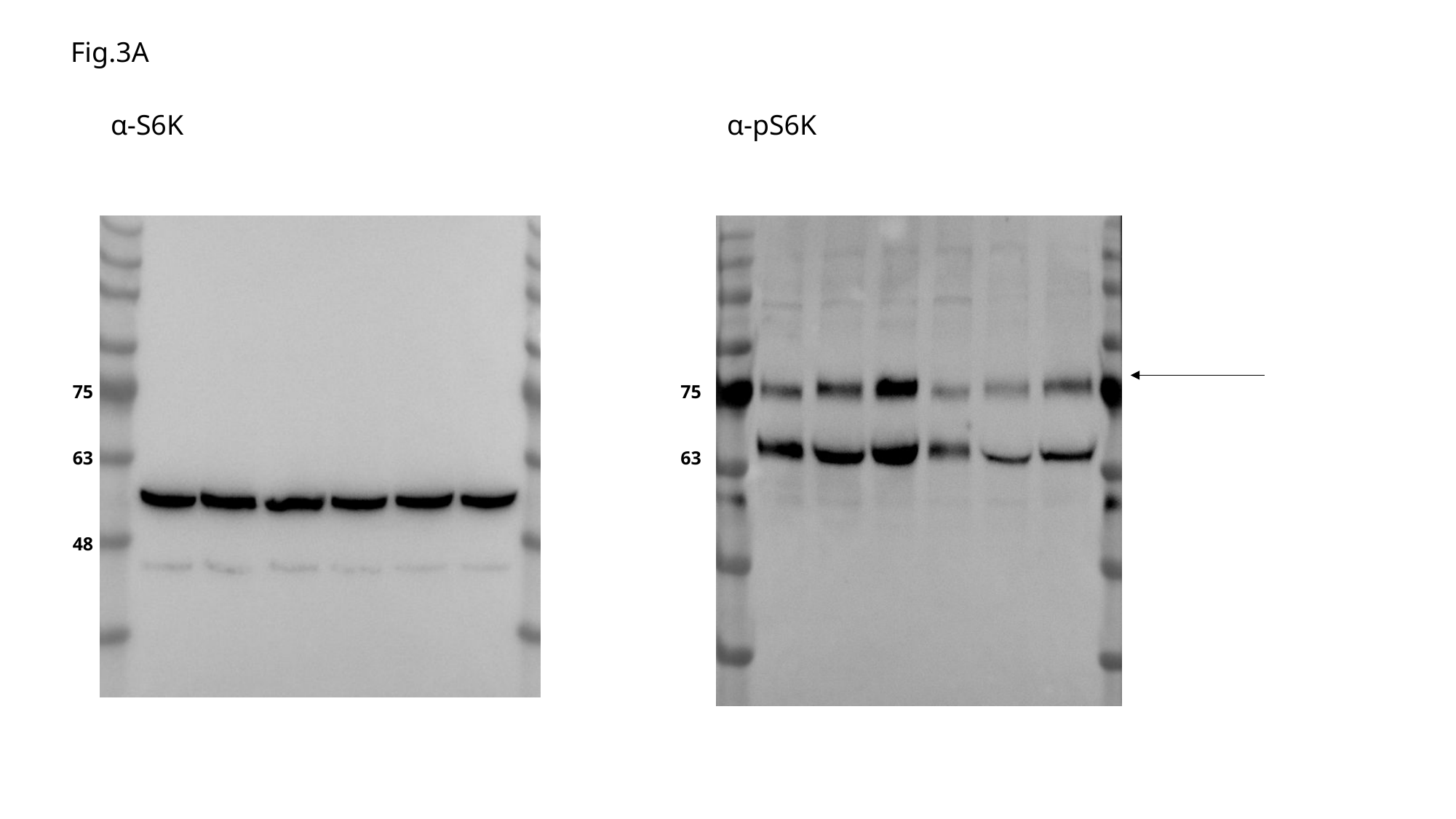

Fig.3A
α-S6K
α-pS6K
75
75
63
63
48

## Slide 4
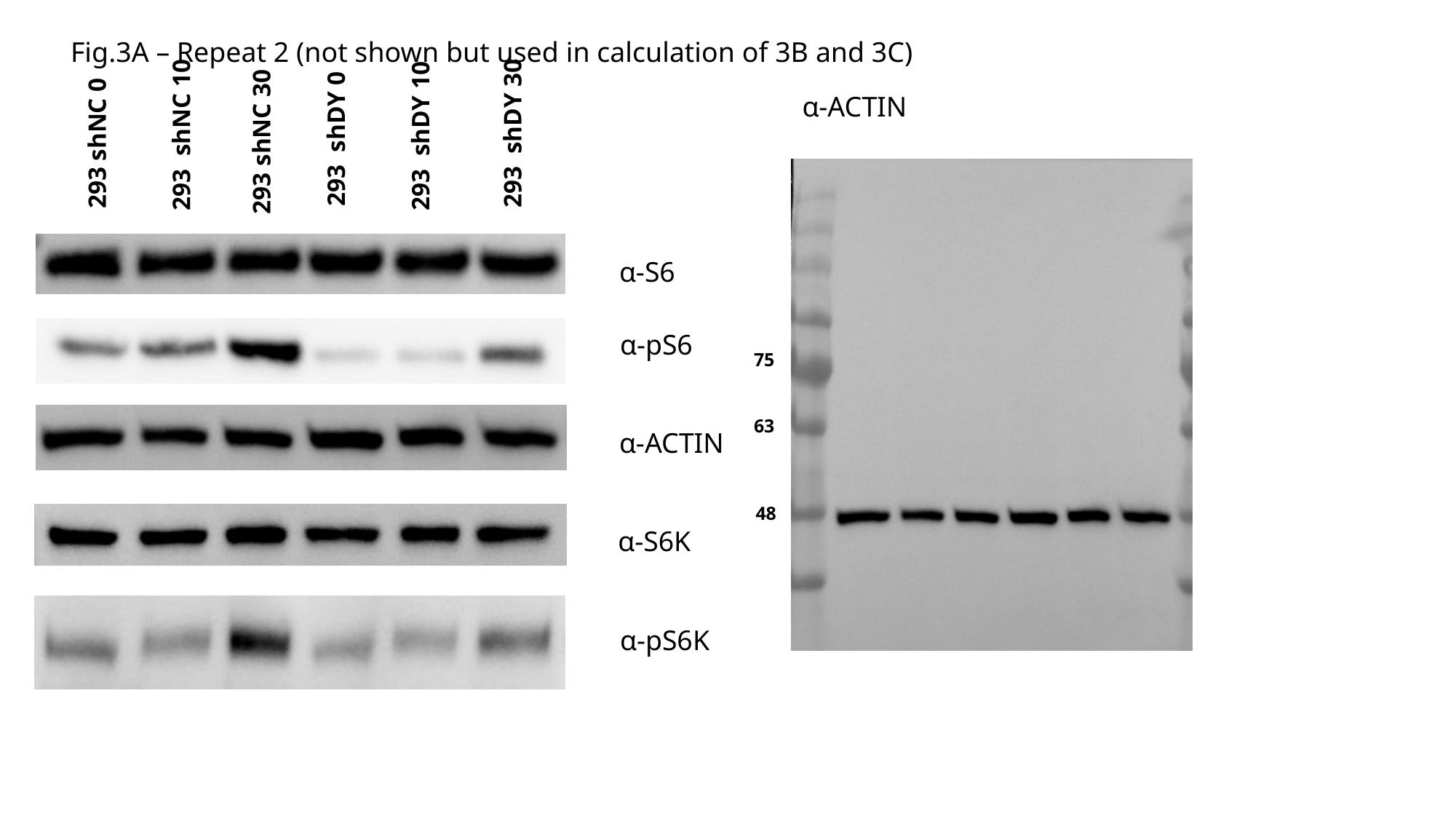

Fig.3A – Repeat 2 (not shown but used in calculation of 3B and 3C)
α-ACTIN
293 shDY 0
293 shNC 0
293 shDY 30
293 shNC 10
293 shDY 10
293 shNC 30
α-S6
α-pS6
75
63
α-ACTIN
48
α-S6K
α-pS6K

## Slide 5
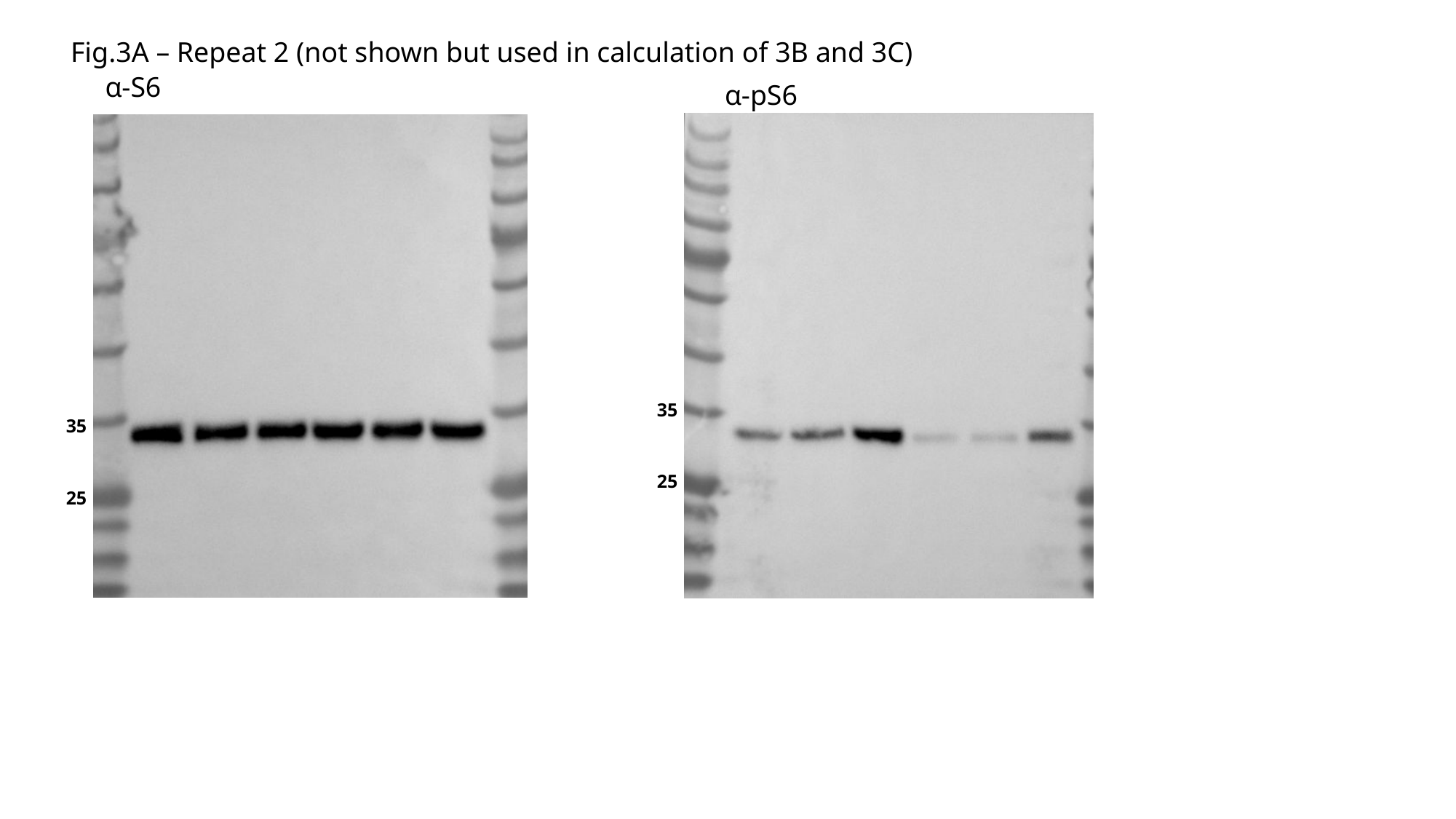

Fig.3A – Repeat 2 (not shown but used in calculation of 3B and 3C)
α-S6
α-pS6
35
35
25
25

## Slide 6
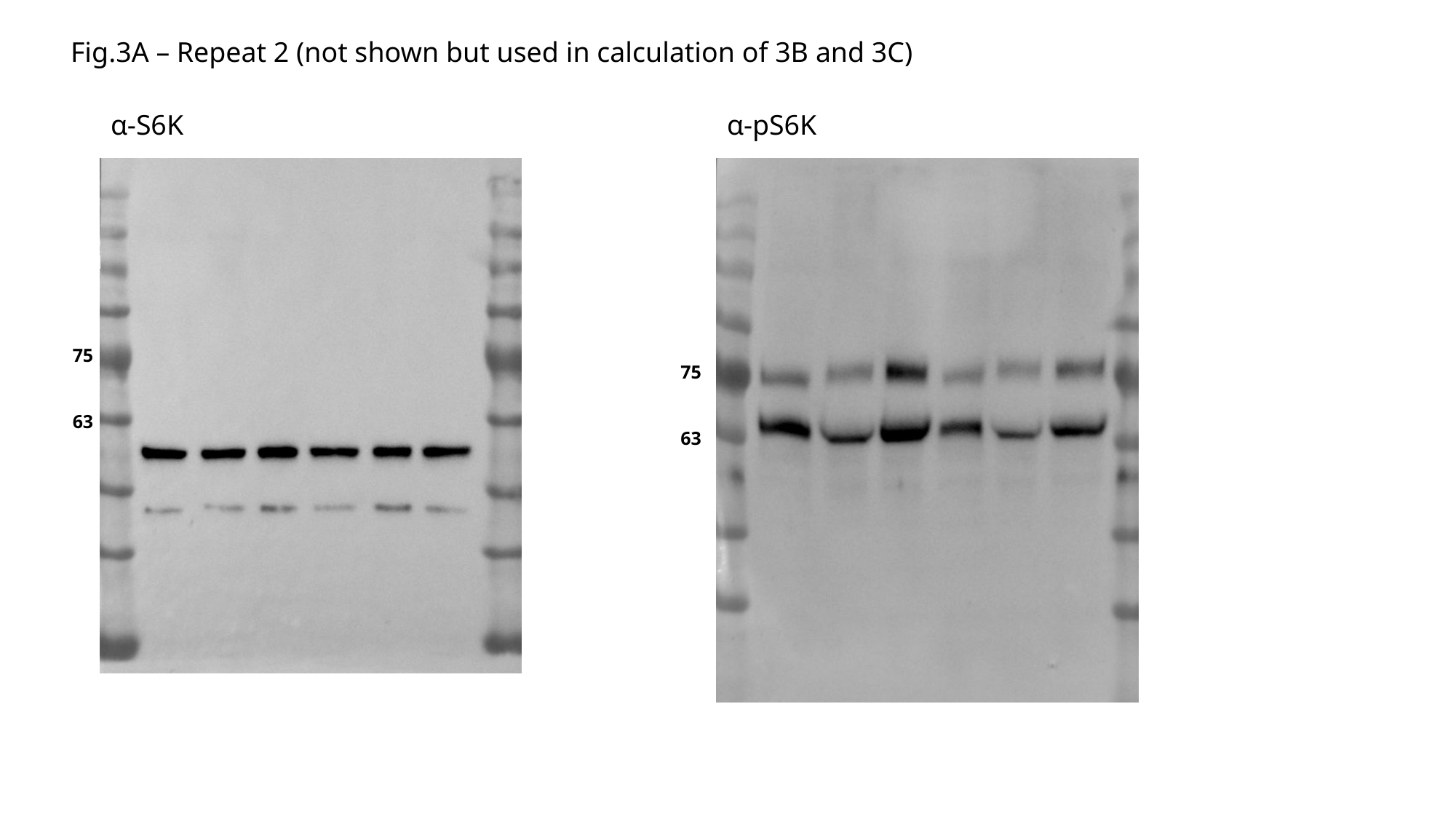

Fig.3A – Repeat 2 (not shown but used in calculation of 3B and 3C)
α-S6K
α-pS6K
75
75
63
63

## Slide 7
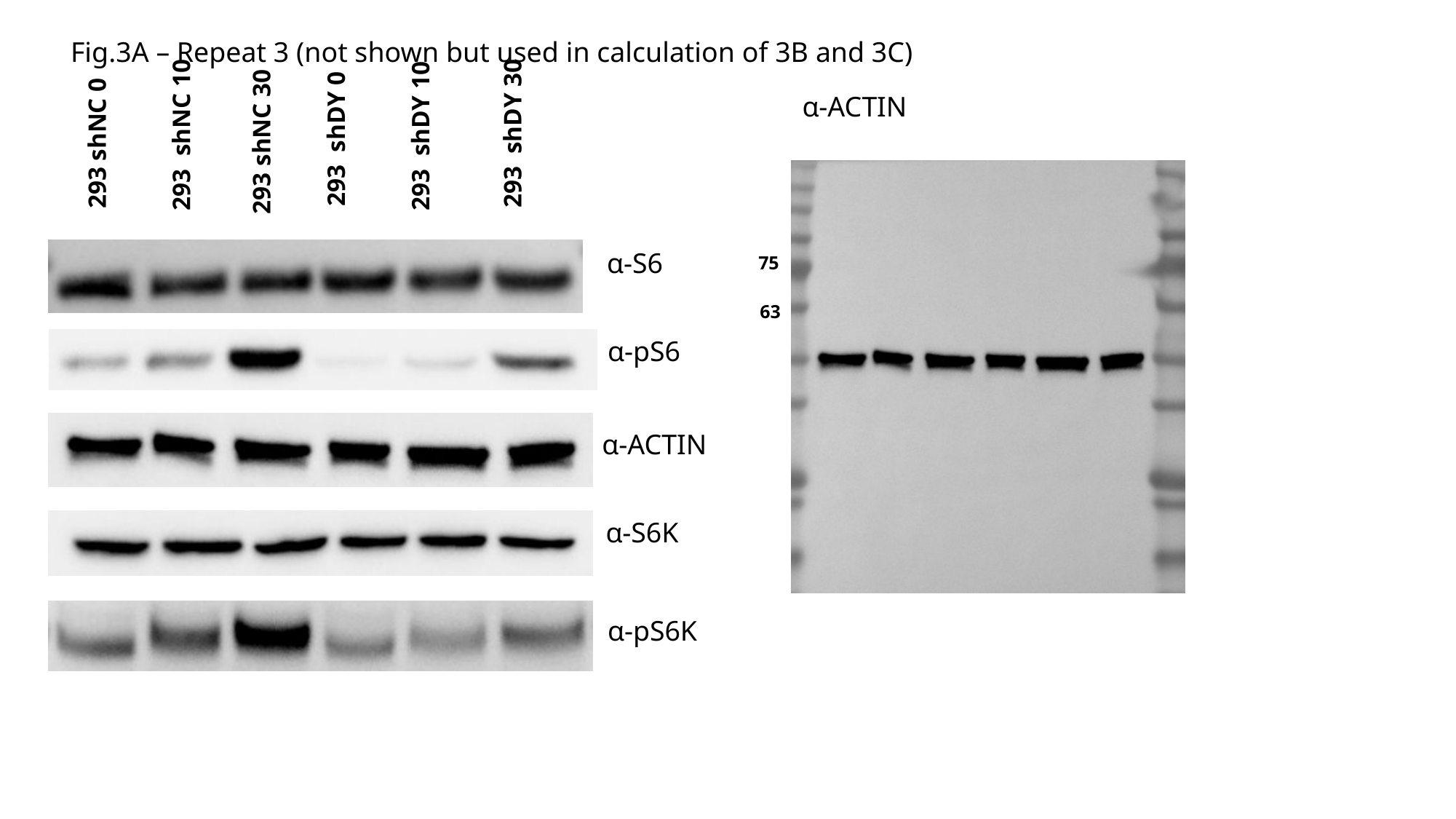

Fig.3A – Repeat 3 (not shown but used in calculation of 3B and 3C)
α-ACTIN
293 shDY 0
293 shNC 0
293 shDY 30
293 shNC 10
293 shDY 10
293 shNC 30
α-S6
75
63
α-pS6
α-ACTIN
α-S6K
α-pS6K

## Slide 8
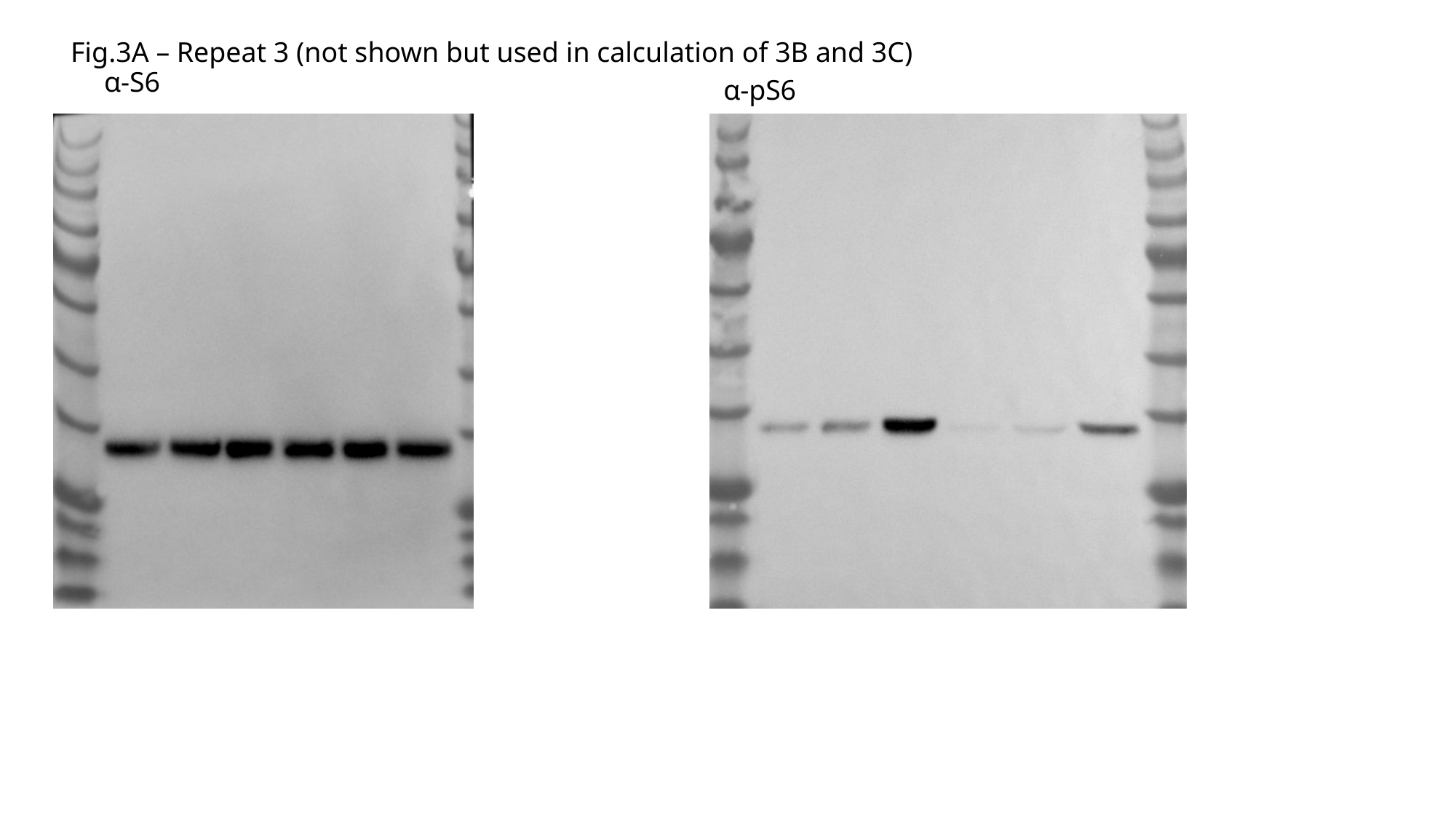

Fig.3A – Repeat 3 (not shown but used in calculation of 3B and 3C)
α-S6
α-pS6

## Slide 9
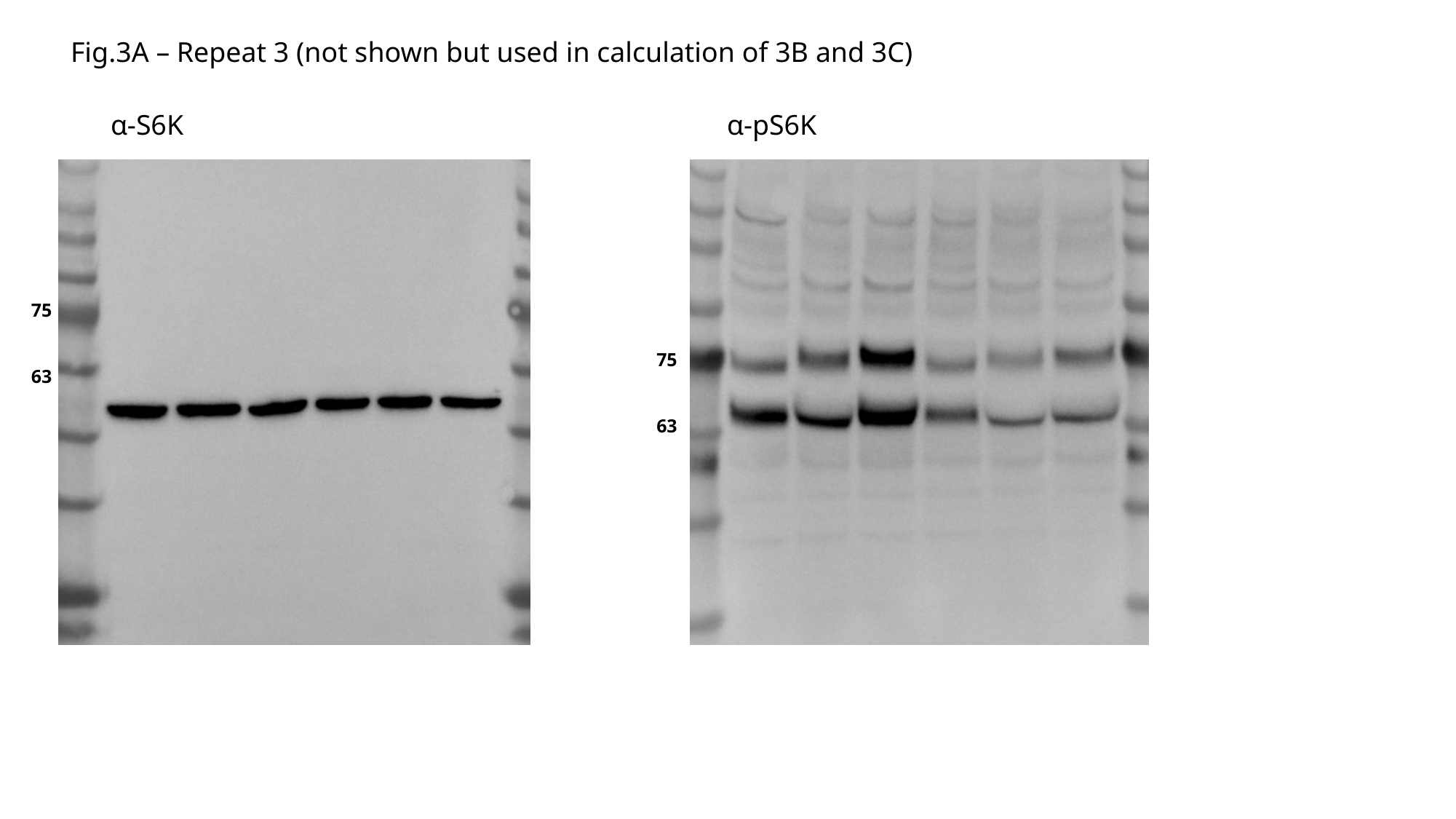

Fig.3A – Repeat 3 (not shown but used in calculation of 3B and 3C)
α-S6K
α-pS6K
75
75
63
63
