# Supplementary material for: DYRK1A interacts with the tuberous sclerosis complex and promotes mTORC1 activity
Source: eLife. 2024 Oct 22;12:RP88318. doi: 10.7554/eLife.88318 (PMC11495841; doi:10.7554/eLife.88318)
Supplement: Figure 3—source data 3. [file elife-88318-fig3-data3.zip › Figure 3D-source data.pptx]

## Slide 1
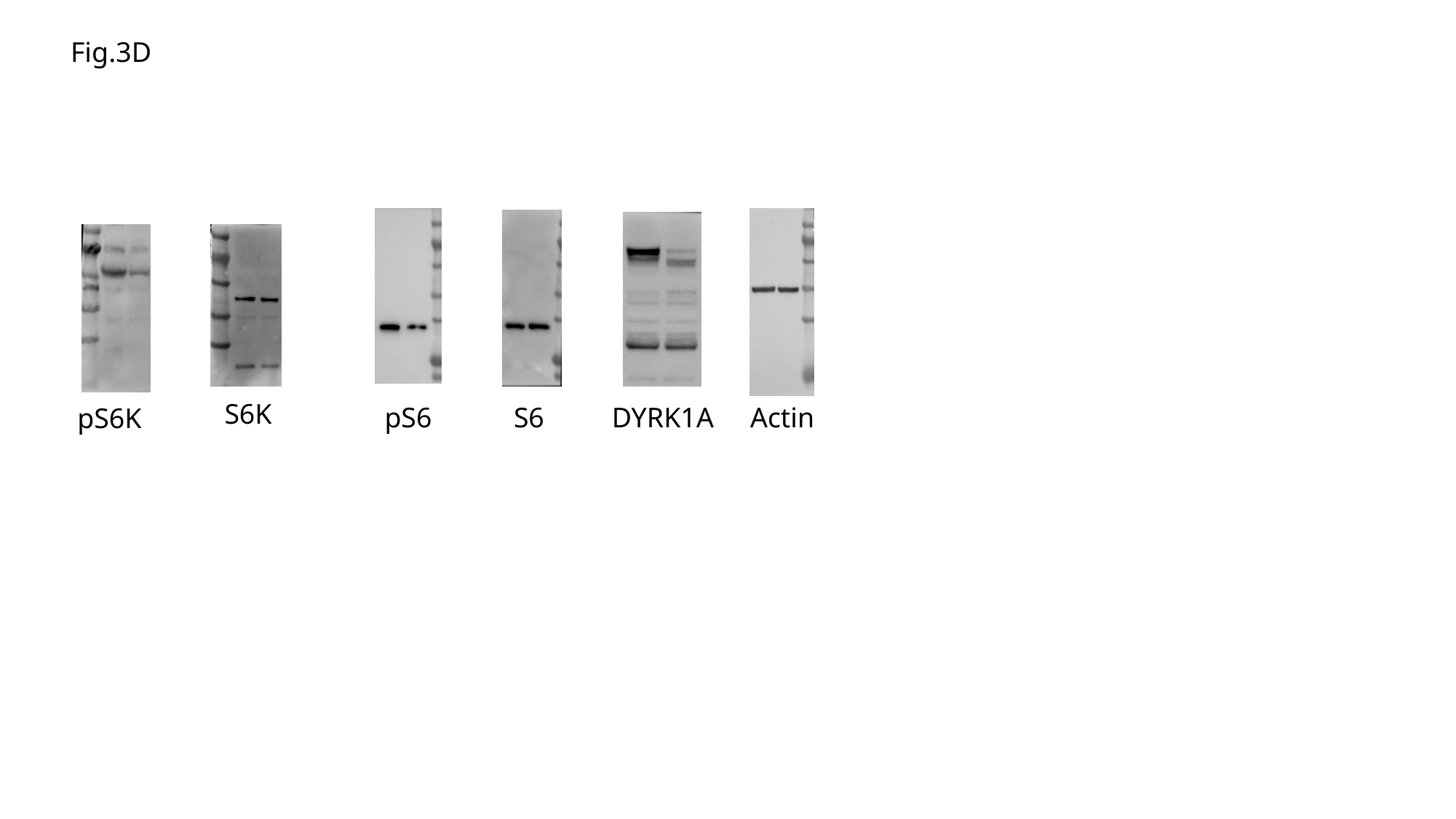

Fig.3D
S6K
DYRK1A
S6
pS6
Actin
pS6K

## Slide 2
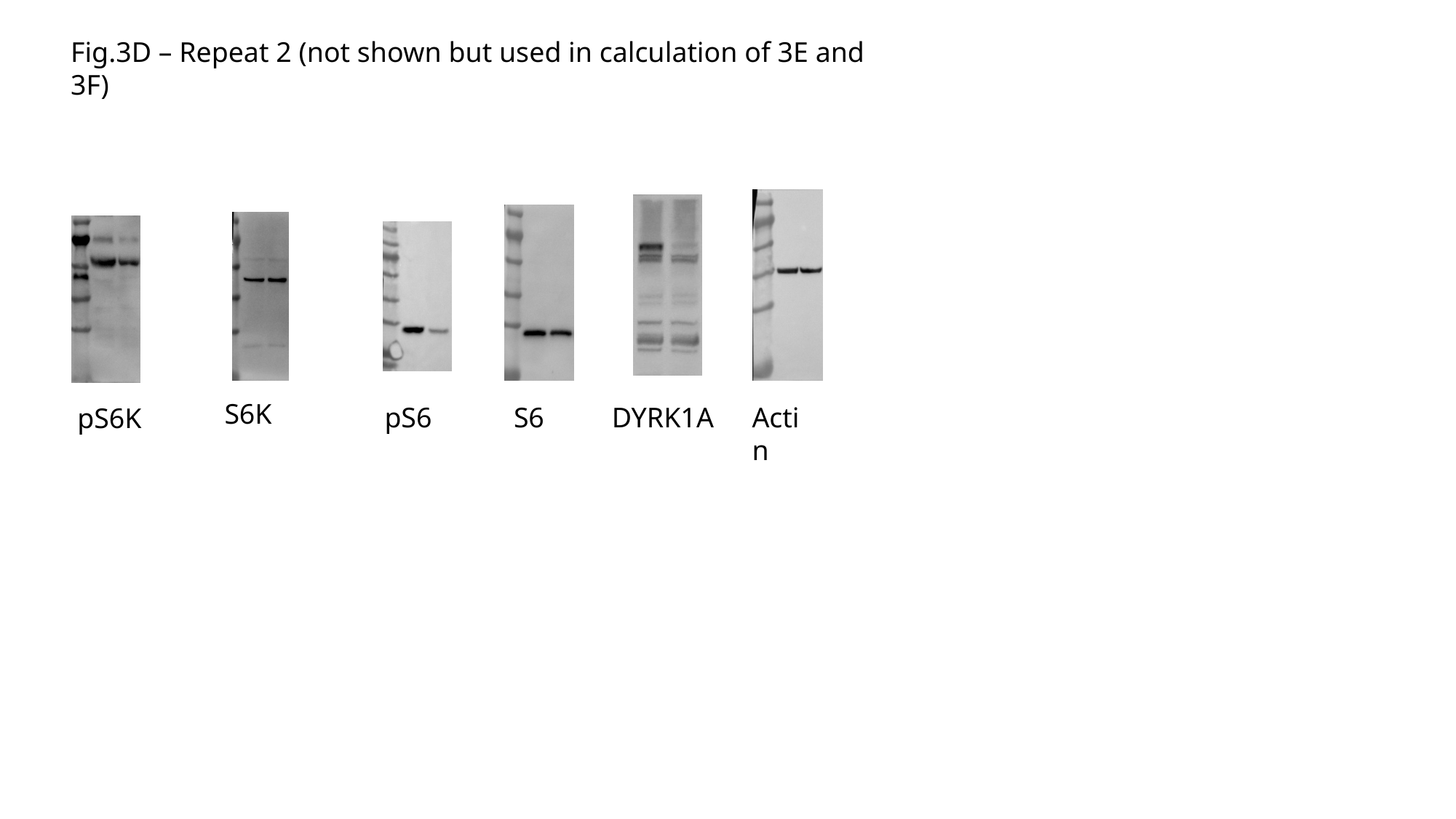

Fig.3D – Repeat 2 (not shown but used in calculation of 3E and 3F)
S6K
DYRK1A
S6
pS6
Actin
pS6K

## Slide 3
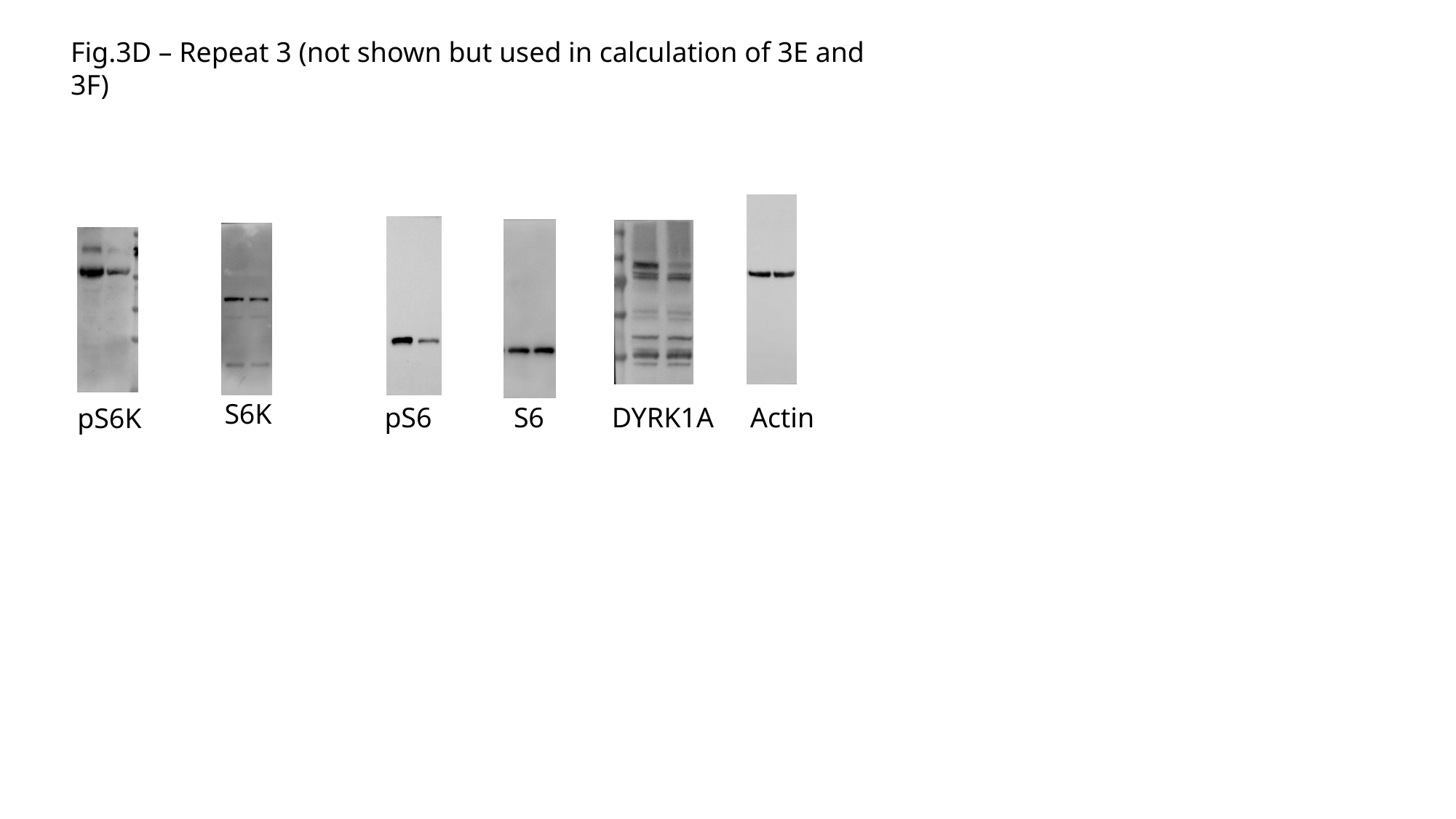

Fig.3D – Repeat 3 (not shown but used in calculation of 3E and 3F)
S6K
DYRK1A
S6
pS6
Actin
pS6K
