# Supplementary material for: DYRK1A interacts with the tuberous sclerosis complex and promotes mTORC1 activity
Source: eLife. 2024 Oct 22;12:RP88318. doi: 10.7554/eLife.88318 (PMC11495841; doi:10.7554/eLife.88318)
Supplement: Figure 4—source data 1. [file elife-88318-fig4-data1.zip › Figure 4A-source data.pptx]

## Slide 1
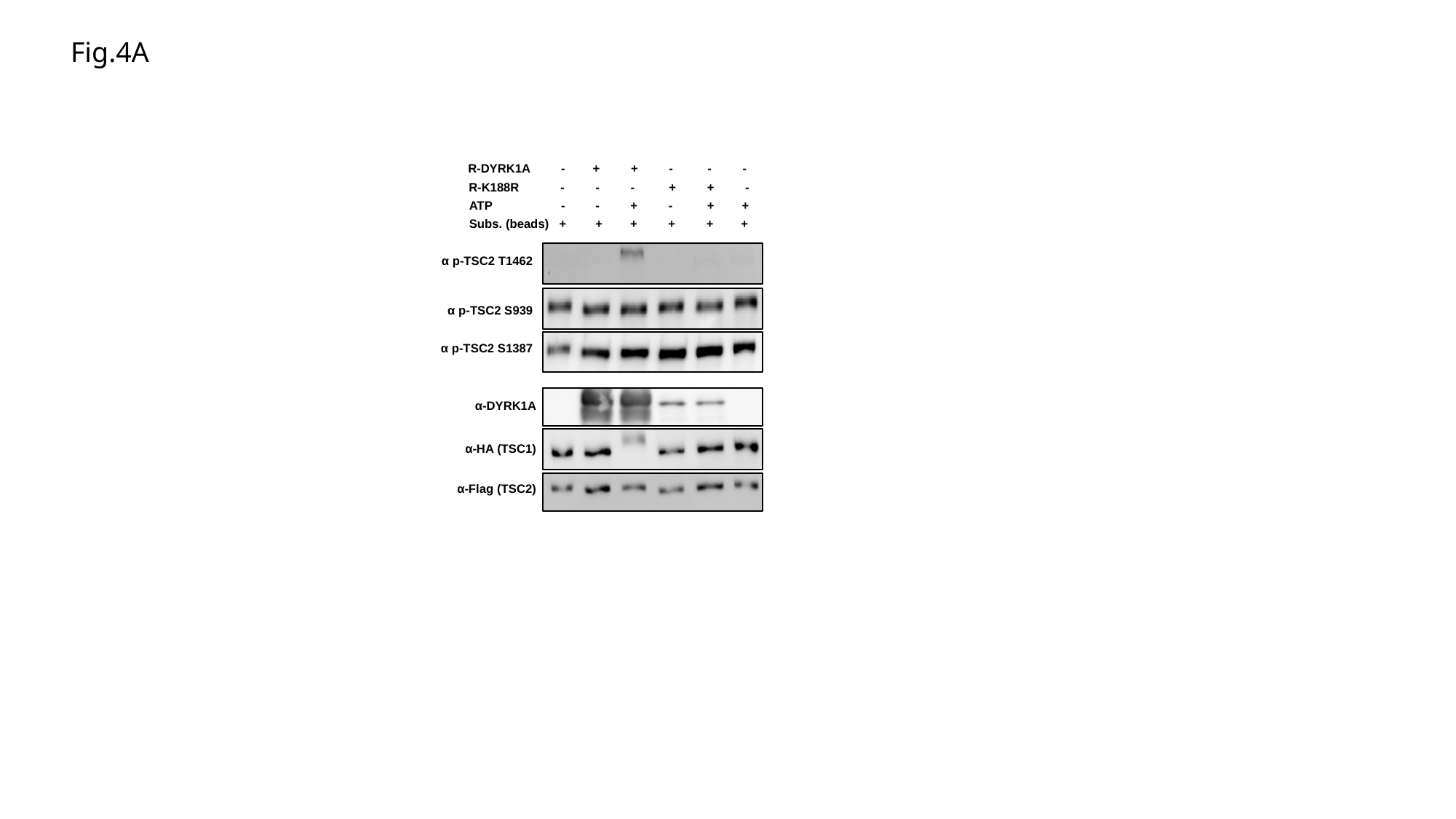

Fig.4A
R-DYRK1A - + + - - -
R-K188R - - - + + -
ATP -	 - + - + +
Subs. (beads) +	 + + + + +
α p-TSC2 T1462
α p-TSC2 S939
α p-TSC2 S1387
α-DYRK1A
α-HA (TSC1)
α-Flag (TSC2)

## Slide 2
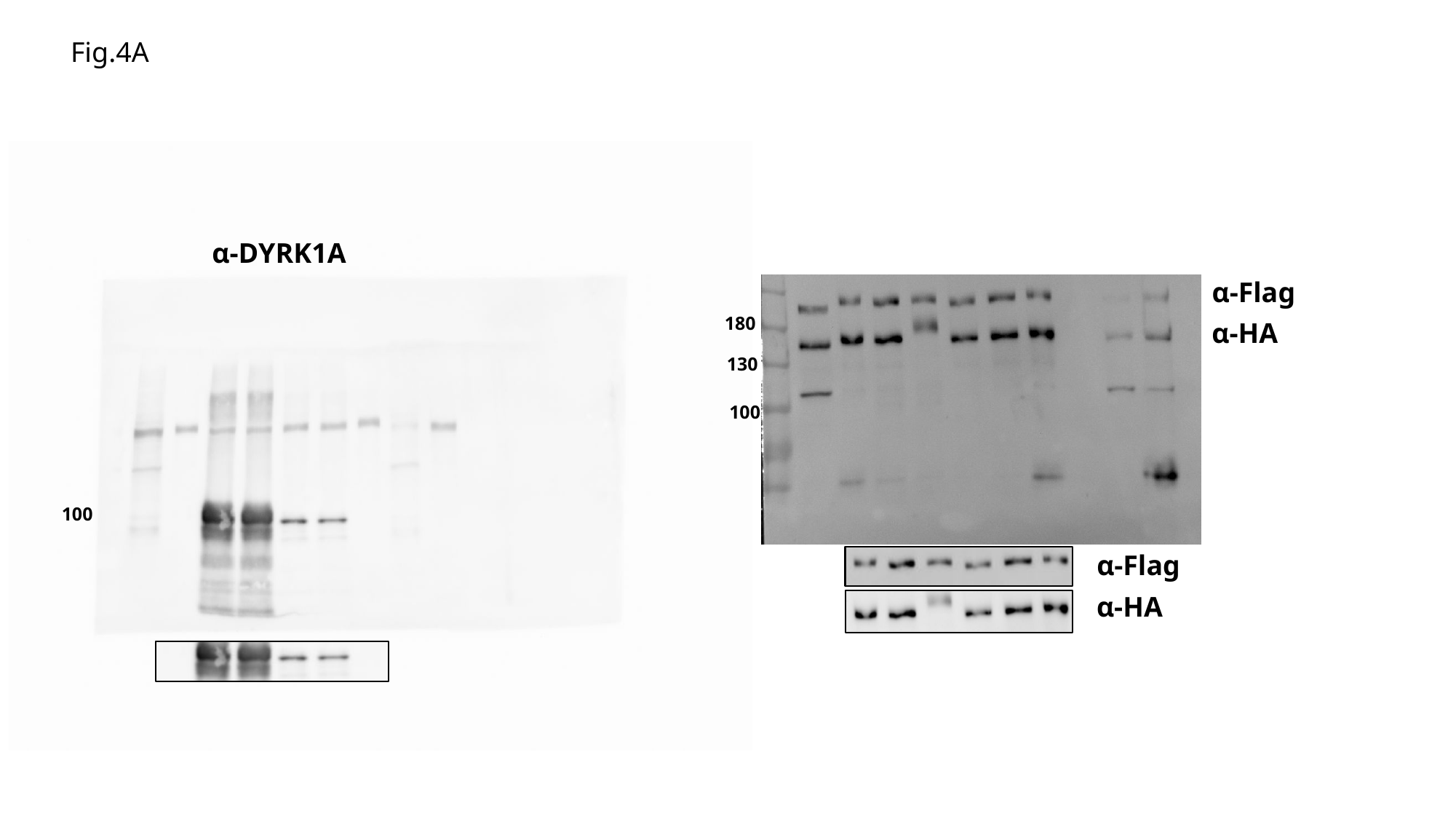

Fig.4A
α-DYRK1A
α-Flag
180
α-HA
130
100
100
α-Flag
α-HA

## Slide 3
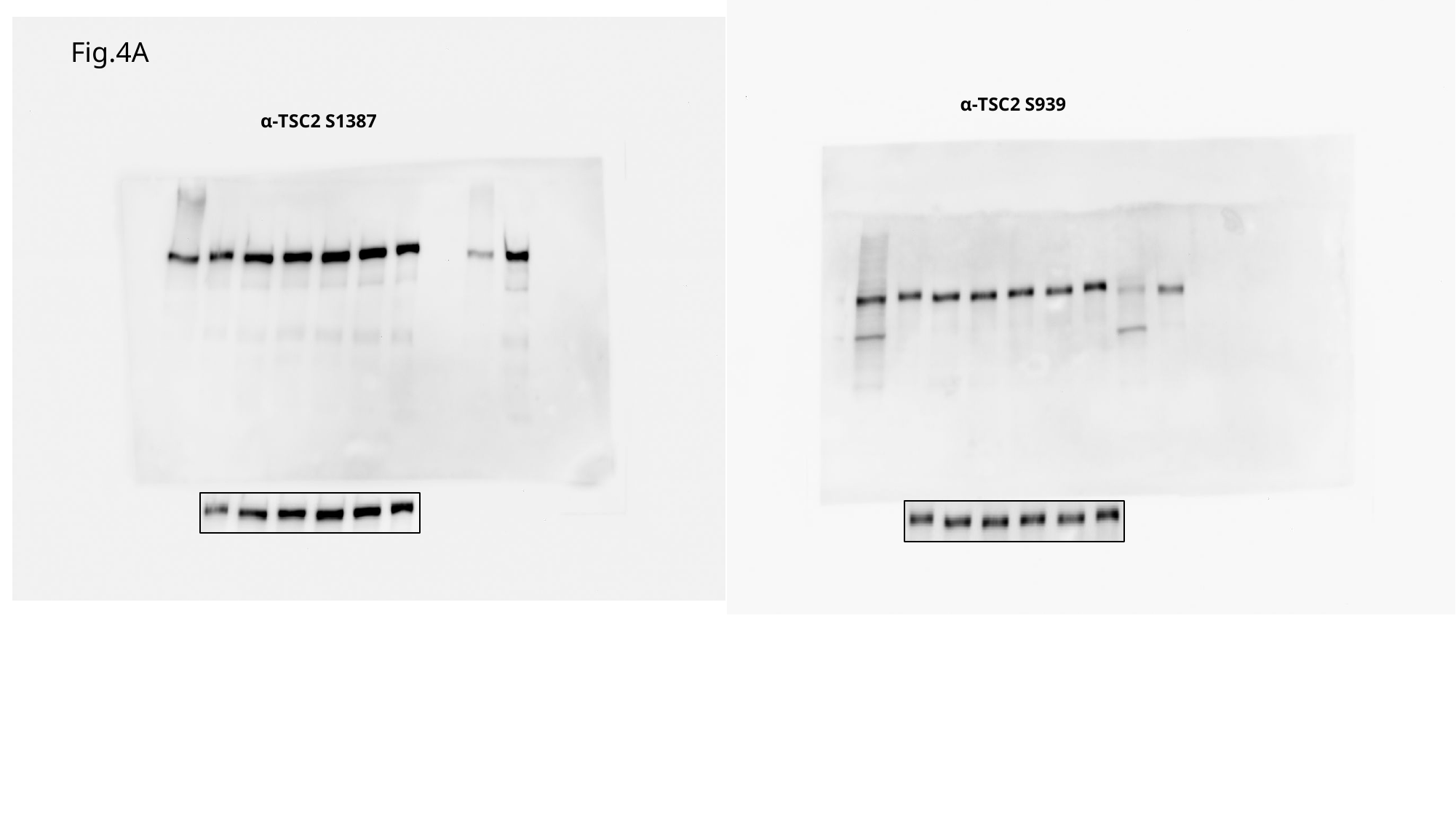

Fig.4A
α-TSC2 S939
α-TSC2 S1387
α-TSC2 S939

## Slide 4
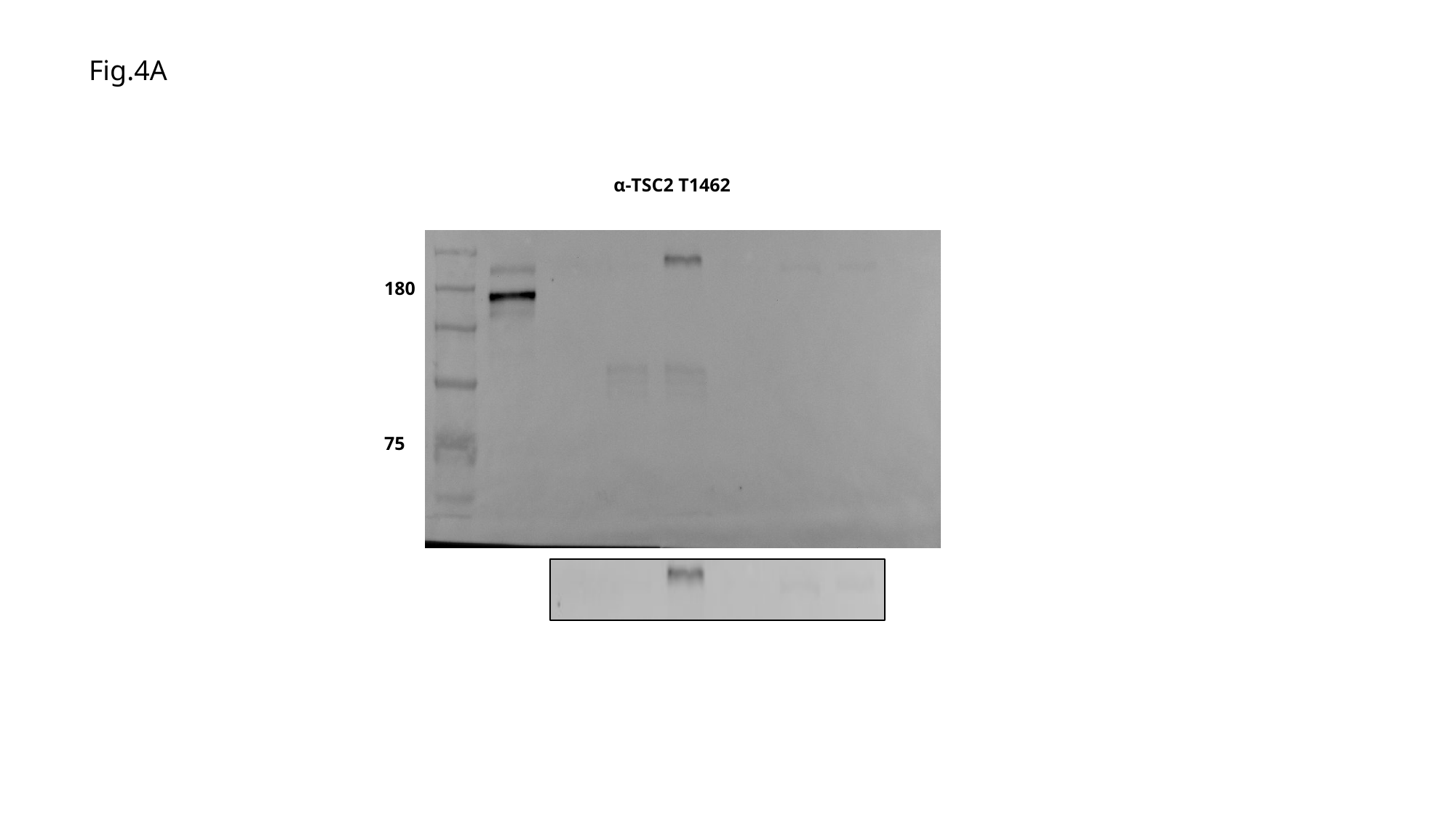

Fig.4A
α-TSC2 T1462
180
75
