# Supplementary material for: DYRK1A interacts with the tuberous sclerosis complex and promotes mTORC1 activity
Source: eLife. 2024 Oct 22;12:RP88318. doi: 10.7554/eLife.88318 (PMC11495841; doi:10.7554/eLife.88318)
Supplement: Supplementary file 1. [file elife-88318-supp1.docx]

**Table S1.** SgRNA target sequences for mouse cells

| Gene name | SgRNA Target |
| --- | --- |
| Control-sgRNA | Gcgaggtattcggctccgcg |
| Dyrk1a-sgRNA1 | gcgcttttatcggtctccag |
